# Stability of SARS-CoV-2 phylogenies

**DOI:** 10.1371/journal.pgen.1009175

**Published:** 2020-11-18

**Authors:** Yatish Turakhia, Nicola De Maio, Bryan Thornlow, Landen Gozashti, Robert Lanfear, Conor R. Walker, Angie S. Hinrichs, Jason D. Fernandes, Rui Borges, Greg Slodkowicz, Lukas Weilguny, David Haussler, Nick Goldman, Russell Corbett-Detig

**Affiliations:** 1 Department of Biomolecular Engineering, University of California Santa Cruz, Santa Cruz, CA, United States of America; 2 Genomics Institute, University of California Santa Cruz, Santa Cruz, CA, United States of America; 3 European Molecular Biology Laboratory, European Bioinformatics Institute (EMBL-EBI), Wellcome Genome Campus, Cambridge, United Kingdom; 4 Department of Organismic and Evolutionary Biology, Harvard University, Cambridge, MA, United States of America; 5 Department of Ecology and Evolution, Research School of Biology, Australian National University, Canberra, ACT, Australia; 6 Department of Genetics, University of Cambridge, Cambridge, United Kingdom; 7 Howard Hughes Medical Institute, University of California, Santa Cruz, CA, United States of America; 8 Institut für Populationsgenetik, Vetmeduni Vienna, Wien, Austria; 9 MRC Laboratory of Molecular Biology, Cambridge, United Kingdom; HudsonAlpha Institute for Biotechnology, UNITED STATES

## Abstract

The SARS-CoV-2 pandemic has led to unprecedented, nearly real-time genetic tracing due to the rapid community sequencing response. Researchers immediately leveraged these data to infer the evolutionary relationships among viral samples and to study key biological questions, including whether host viral genome editing and recombination are features of SARS-CoV-2 evolution. This global sequencing effort is inherently decentralized and must rely on data collected by many labs using a wide variety of molecular and bioinformatic techniques. There is thus a strong possibility that systematic errors associated with lab—or protocol—specific practices affect some sequences in the repositories. We find that some recurrent mutations in reported SARS-CoV-2 genome sequences have been observed predominantly or exclusively by single labs, co-localize with commonly used primer binding sites and are more likely to affect the protein-coding sequences than other similarly recurrent mutations. We show that their inclusion can affect phylogenetic inference on scales relevant to local lineage tracing, and make it appear as though there has been an excess of recurrent mutation or recombination among viral lineages. We suggest how samples can be screened and problematic variants removed, and we plan to regularly inform the scientific community with our updated results as more SARS-CoV-2 genome sequences are shared (https://virological.org/t/issues-with-sars-cov-2-sequencing-data/473 and https://virological.org/t/masking-strategies-for-sars-cov-2-alignments/480). We also develop tools for comparing and visualizing differences among very large phylogenies and we show that consistent clade- and tree-based comparisons can be made between phylogenies produced by different groups. These will facilitate evolutionary inferences and comparisons among phylogenies produced for a wide array of purposes. Building on the SARS-CoV-2 Genome Browser at UCSC, we present a toolkit to compare, analyze and combine SARS-CoV-2 phylogenies, find and remove potential sequencing errors and establish a widely shared, stable clade structure for a more accurate scientific inference and discourse.

## Author summary

SARS-CoV-2 genome sequences have been produced by hundreds of labs across the world. Idiosyncratic data generation or processing has the potential to inject non-random errors into genome sequences provided by individual lab groups. Here we show that these sites can be detected and removed by identifying variants that appear to reoccur many times across a phylogeny and are associated with specific lab groups. We show that the identified variants are consistent with expectations for recurrent error. These sites may produce spurious signatures of natural selection and viral recombination. We also provide methods for comparisons and visualization of extremely large phylogenies.

## Introduction

**Fig 1:**
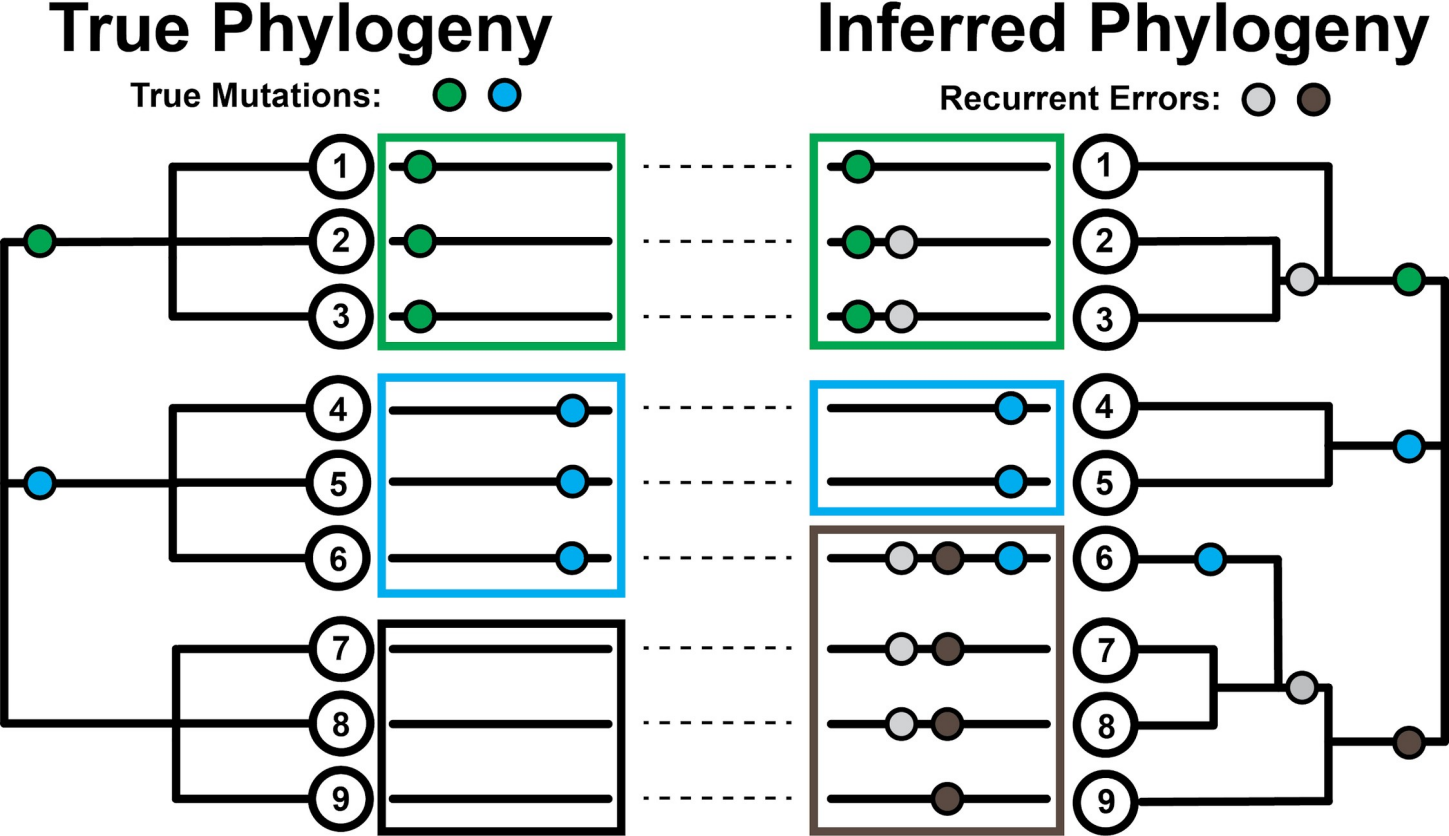
Effect of recurrent sequencing errors on phylogenetic inferences. (Left) Pictorial representation of how the evolutionary histories of viral sequences (long black lines adjacent to tree nodes) can be traced on a phylogenetic tree using mutational events (green and blue circles). In this case, each mutation occurs once independently. (Right) The introduction of recurrent errors (gray and brown circles) can obscure the true evolutionary relationship between sequences leading to the inference of artifactual subgroups/clades (green-gray, leaves 2 & 3, and gray-brown, leaves 7 & 8) and even the incorrect assignment of viral sequences to subgroups (leaf 6 no longer correctly groups with the blue subgroup containing leaves 4 & 5). Large boxes group together subgroups based on inferred first mutation. Note that systematic errors must be non-heritable and their inferred placement on internal branches reflects their impacts on phylogenetic inference. We display this example as ‘clock-like’ for additional clarity.

Extremely rapid whole-genome sequencing has enabled nearly real-time tracing of the evolution of the SARS-CoV-2 pandemic [1–6]. By leveraging sequence data produced by labs throughout the world, researchers can trace the transmission of the virus across human populations [7–15]. Typically, viral evolution is encapsulated by a phylogenetic tree relating all of the virus samples in a large set to one another [6,16–20]. However, despite efforts to mitigate the impact of sequencing and assembly errors, and to provide standardized datasets for real-time analysis [21], inferred phylogenetic histories of the outbreak often differ between analyses of different research groups (Results) and these inferred histories sometimes differ between analyses performed by the same group with different data (*e*.*g*., 31 different Nextstrain trees produced between 3/23 and 4/30: Results). These differences may be created or accentuated when samples that contain unidentified sequencing errors are incorporated into the phylogenetic tree. Defining stable and easily referenced major clades of the virus is essential for epidemiological studies of viral population dynamics [18,19]. An understanding of how errors might be affecting the trees that are being published is essential to achieving that goal (Fig 1).

It can be difficult to distinguish sequencing errors of different types from genuine transmitted and non-transmitted mutations in genome sequences. Taking a conservative approach, many researchers remove variants that are observed only once during the evolution of the virus when constructing a phylogenetic tree, as these may be more likely to be errors [22,23] or non-transmitted mutations. However, systematic errors, where the same error from a common source is introduced many times in otherwise distinct viral genome sequences, are not removed by that approach [24,25]. These are more problematic, as they can appear as if they are genuine transmitted mutations (Fig 1). This might result from recurring errors in data generation or processing, or due to contamination among samples. Each case induces an apparent mutation that may be challenging to reconcile with the real structure of the viral tree. Consequently, systematic errors can produce support for erroneous relationships between viral isolates and destabilize tree-building efforts. One possible approach is to mask out specific sites in the genome sequence where recurring errors are suspected, as suggested previously [24]. However, genuine recurrent mutations that may contain important information about properties of viral evolution [7,9,26–28] are sometimes hard to distinguish from recurrent systematic errors, and this could obscure important biology. Here, we present results that we hope will help the community make the critical decision as to how to identify and treat potential errors in SARS-CoV-2 genome sequences.

In addition to their influence on phylogenetic inference, systematic errors can also lead to erroneous inferences about viral mutation processes, recombination and selection. For example, artefactual biases in mutational processes could confound signatures of mutational hotspots [29–34]. The issue of whether or not recombination has occurred during the outbreak is critical to the immunological battles against the virus and is under intense debate [7,35–41]. Because many tests of recombination assume that all mutations can only occur once at each site, recurrent mutation and systematic errors can confound signatures of recombination [7,27,36]. Finally, recurrent mutations have been identified as a possible signature of elevated mutation rates or natural selection in SARS-CoV-2 [9,14,24,26,27,30,34,36,42], but some of these apparent instances of selection may be due to systematic errors in the sequences. Confusion about recurrent mutations and recombination affects our understanding of host response and influences our decisions about which viral molecular processes or specific immune epitopes we might want to target in vaccine development. Thus, it is essential that we explore the possible extent and impact of systematic errors in the viral genome sequences.

Another basic problem in current investigations of viral evolution is widespread phylogenetic uncertainty. In part, this has prevented the community from settling on a consensus definition of distinct viral clades (“(sub)types”, “groups”, “lineages”) representing the early divergence events, producing communication problems in the scientific discourse [18,43]. Furthermore, many groups are making phylogenetic trees with widely varying goals, including dissecting patterns of nucleotide substitution, recurrent mutation, local lineage tracing, and large-scale phylogenomics [9,18,27]. The resulting topologies vary dramatically in structure, owing to differences in analysis choices and to phylogenetic uncertainty stemming from limited genetic diversity in the expanding viral populations [44]. Consistent approaches for identifying commonalities and rectifying differences among trees are therefore foundational to the efforts to characterize viral evolution and epidemiology. A maximally stable topology will be essential for consistent nomenclature and facilitating conversations between analyses [18,43].

Our work takes on these two interrelated subjects: systematic errors and phylogenetic uncertainty. First, we show that hundreds of samples in the current SARS-CoV-2 sequencing datasets are affected by lab-associated variants, which are potentially erroneous. These variants distort phylogenetic inferences at scales most relevant to local lineage tracing and impact inferred patterns of mutational recurrence and recombination. We demonstrate that many can be identified and removed by cross-referencing patterns of recurrence against the source sequencing lab, and we provide automated methods for detecting suspicious and highly recurrent mutations. Furthermore, we intend to regularly update our methods and results, and share our current inferred errors and alignment masking recommendations with the scientific community through the virological.org website (specifically posts https://virological.org/t/issues-with-sars-cov-2-sequencing-data/473 and https://virological.org/t/masking-strategies-for-sars-cov-2-alignments/480). Second, to facilitate communication and comparison across different SARS-CoV-2 phylogenies, we develop approaches for efficiently comparing and visualizing differences among trees. All of the tools and functionality that we describe here are publicly available and integrated into the UCSC Genome Browser to facilitate rapid visualization, data exploration, and cross-referencing among datasets and analyses. We anticipate that these methods will fuel more accurate continued discovery during the current pandemic and beyond.

## Results/discussion

### Nextstrain datasets

Our analyses are built in large part on the work of Nextstrain [16]. This team has already implemented a number of precautions to remove problematic sites and samples. In particular, they remove samples that are too divergent from others or whose date of sampling is inconsistent with the number of accumulated mutations. Additionally, all indels relative to the reference in the resulting multiple alignment are masked. Here, we do not consider the impact of alternative multiple alignments, upstream filtering methods, or the possible impacts of indels. Each of these factors has the potential to affect downstream analyses and should be considered carefully. For our purposes, we anticipate that Nextstrain’s filters will minimize idiosyncratic errors and should be retained in the majority of future analyses. Here, as a primary example we use 31 different alignments and phylogenetic trees inferred by Nextstrain from days between 3/23/2020 and 4/30/2020. We focused in particular on the dataset from 4/19/2020, which contains 3246 variant positions in total (Methods). The vast majority of variants are at low frequency, as is expected for a rapidly expanding population.

### Systematic error could be mistaken for recurrent mutation or recombination

Non-random errors present a fundamental challenge for phylogenetic inference and to the interpretation of viral evolutionary dynamics. These challenges can be particularly severe in cases, like that of SARS-CoV-2, that involve large collections of sequences with very limited phylogenetic signal [44]. There are at least four possible sources of (real or apparent) variants that recur within independent lineages in a tree, and each should be accompanied by a distinct pattern (Table 1). In particular, recent work has shown a strong bias towards C>U mutation in the SARS-CoV-2 genome [22,43,45,46]. Systematic errors, which usually result from consistent errors in molecular biology techniques or bioinformatic data processing, are subject to biases that are of no biological relevance and can confound the inference of mutation rates. Such errors can also give the appearance of elevated evolutionary rate at some sites which may be mistaken for a signature of positive selection. We therefore anticipate that many systematic errors will affect many mutation types, modify protein sequences, and strongly correlate with genome sequences generated in particular labs [24].

**Table 1:** Expectations for various sources of apparent recurrent mutation. *As defined in the main text (below). **Owing to ours and previous works, we expect that most recurrent mutations will usually demonstrate a C>U bias; however, this may not be uniformly the case for example in mutation hotspots.

Source	Heritable	Typical Allele Frequency	C>U Biased	Lab Correlation	Extremal*
Recurrent Mutation	Y	Low-Moderate	Y**	N	Y
Recombination	Y	High	Possible	N	N
Systematic Error	N	Low	Possible	Y	Y
Contamination Error	N	High	N	Possible	N

### Many apparently recurrent mutations found in the SARS-CoV-2 genome

**Fig 2:**
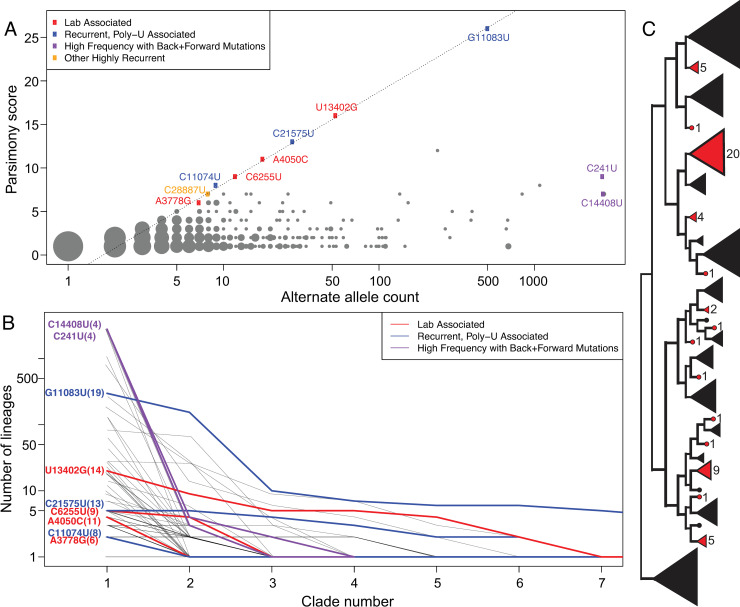
**(A) The relationship between alternate allele count and parsimony score.** Point radius indicates how many sites share a single parsimony score and alternate allele count. Several noteworthy recurrent mutations are labelled. Note that the X-axis is log-scaled. (B) The sizes of independent clades for the same alternate allele arranged in descending order. The number of lineages per clade is shown on logarithmic scale facilitating comparison with Panel (A). These indicate that when alternate allele clade sizes for a given site are sorted in decreasing order, their sizes are reduced going from left to right by a multiplicative factor at each step, consistent with the log-linear relationship displayed in Panel (A). Variants with remarkably high recurrence are shown with color reflecting their properties: lab-associated (red), recurrent and associated with a poly-U stretch (blue), and high frequency with many forward and backward mutations (purple). Grey lines in the background are the same values but for all other variants with parsimony score 4 or greater. The values in parentheses in the variant names indicate the number of unique clades associated with the alternate allele. Note that in some cases, this extends beyond the limit of the X-axis and that the Y-axis is log-scaled for visibility. (C) An example of the observed patterns of evolution at one highly recurrent site with reference allele U and alternate allele G, site 13402 and parsimony score 14, where 14 alternate allele clades (in red) each represent an apparently independent incidence of the mutation substituting the alternate allele.

To examine patterns of recurrent mutation we employ a simple statistic, the parsimony score, which is the count of the minimum number of unique mutation events consistent with a tree and sample genotypes ([47,48], computed using our software from https://github.com/yatisht/strain_phylogenetics, Methods). More sophisticated statistics could be employed, but this simple one is effective, is readily interpretable, and can be computed rapidly. We restrict most analyses to bi-allelic sites, i.e. sites that contain the allele in the reference genome from the root of the tree (here and in Nextstrain this is sample Wuhan/Hu-1, obtained in December 2019 in the city of Wuhan, China, RefSeq accession NC_045512.2) and a single alternate allele. At the time of these analyses, multiallelic sites (with >1 alternate alleles) were sufficiently rare so that this did not significantly affect the analyses; however, in our more recent analyses (see [24]) we relaxed this assumption. Across the 4/19/2020 Nextstrain tree, we found 2533, 395, 94, 40, and 44 bi-allelic sites with parsimony score 1, 2, 3, 4, or 5 or more, respectively (Fig 2, S1 Fig). In particular, there is a strong “on diagonal” component of the data that is defined by a linear relationship between the log of the alternate allele count and parsimony score (dashed line in Fig 2A, log2-based slope = 3.188). These mutations reoccur across the phylogeny at exceptional rates relative to their allele frequencies. Hereafter, we refer to the set of variants in this on-diagonal group as extremal sites (blue, red, and orange in Fig 2A). This relationship suggests that the extreme accumulation of independent clades for the alternate allele is logarithmically related to the number of instances of the alternate allele in the phylogeny (Fig 2B). While this is in line with the expectation that multiple recurrences of the same substitution would increase the overall alternate allele count, another possible explanation for some of these sites is that apparent variants caused by systematic errors would cause samples with such errors to be wrongly inferred to group together in clades of various sizes (see e.g. Fig 1).

### Automated detection of extremal sites

We sought to provide researchers with a method for rapidly identifying and flagging suspiciously recurrent mutations. We therefore developed code to identify the “on diagonal” extremal sites and produce plots of the output similar to Fig 2, available at https://github.com/yatisht/strain_phylogenetics. Note that depending on the dataset, this component is not always so linear as in Fig 2, but it is associated with highly homoplastic sites regardless (*e.g., S1 Fig*). In itself, this analysis does not differentiate between likely hypermutable sites and systematic errors. Our list of extremal sites includes two that we later show are strongly lab-associated (A4050C and U13402G, likely to be systematic errors), three variants that are adjacent to >5bp poly-U segments in the genome (C11074U, G11083U, and C21575U), as well as two more C>U variants (C21711U, C28887U). Regardless of their proximate causes, highly recurrent mutations can negatively impact the accuracy of inferred tree topologies, and thus we recommend their removal prior to phylogenetic tree construction and many subsequent analyses.

### SARS-CoV-2 data contains many lab-associated variants

To search for systematic errors associated with a particular lab, we extracted the set of sites with parsimony score 4 or more. We then flagged sites as lab-associated variants if more than 80% of the samples containing the alternate allele were generated by a single group. Using this heuristic approach, we found 16 such sites (S1 Table). We note that this set of sites contains two variants previously identified as lab-associated [24], some others identified as highly homoplastic [9,24,26,43], as well as several identified as evidence for recombination [27]. These lab-associated sites display a range of base compositions and only one is a C>U transition (C6255U). This proportion of C>U variants is much less than expected based on the genome-wide average proportion of non-singleton C>U variants (49%, P < 0.0005, Fisher’s exact test), and differs significantly from the proportion of C>U variants among our set of inferred highly recurrent mutations that are not strongly associated with a single sequencing lab (P ≈ 10^−7^, Fisher’s exact test). Furthermore, our set of lab-associated variants is weakly enriched for protein-altering mutations relative to other highly recurrent mutations (P = 0.094). Collectively, our results suggest that many of these 16 lab-associated variants could be systematic errors rather than the result of mutation events.

The potential causes of lab-associated variants are numerous. A non-exhaustive list follows. First, primers for reverse transcription or PCR might introduce systematic errors either via errant priming, because they “overwrite” true variation, or because of errors during bioinformatic processing. For example, the commonly used ARTIC primer sets amplify the viral genome from metatranscriptomic cDNA by tiling the viral genome with PCR amplicons (https://artic.network/). Second, if a portion (perhaps a single amplicon) from a contaminating sample were present in many sequencing reactions from a single lab, this could propagate variants across all genome sequences from a single group. Third, contamination from the human transcriptome itself might be inadvertently included in assembled viral genomes. Finally, RNA degradation during sample handling or processing might be the source of lab-associated variants (Torsten Seemann, comm. at https://virological.org/t/gained-stops-in-data-from-the-peter-doherty-institute-for-infection-and-immunity/486/8).

**Fig 3:**
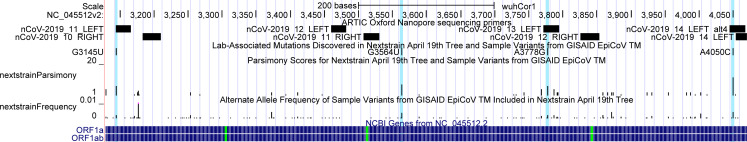
UCSC Genome Browser display of lab-associated variants and ARTIC primers. Bases 3130 to 4070 of the SARS-CoV-2 genome are displayed, containing four lab-associated variants highlighted in light blue. G3145U, A3778G and A4050C overlap ARTIC primer bind sites. An interactive view of this figure is available from http://genome.ucsc.edu/s/SARS_CoV2/labAssocMuts.

Two labs contributed a disproportionate number of lab-associated variants in our dataset, suggesting a consistent source of these alternate alleles (S1 Table). One lab group is strongly associated with two adjacent high parsimony scores and perfectly linked variants A24389C and G24390C. These occur in a 10bp sequence in the genome, CAGCAAGTT, that otherwise closely resembles an Oxford Nanopore sequencing adapter, CAGCACCTT, and is adjacent to an ARTIC primer binding site. Here, the differences between the genome sequence and adapter are bolded. See also [24], where a commenter on that work comes to a similar conclusion regarding the likely source of these variants. Additionally, A4050C, U8022G, U13402G, and A13947U (Fig 2, S1 Table) are associated with this same lab and either overlap or are within 10bp of ARTIC primer binding sites (14_left_alt4, 26_right, 44_right, and 47_left, respectively), suggesting that a consistent bioinformatics data processing error may be responsible. Sequences submitted by another lab group are strongly associated with four additional high parsimony score variants, G2198A, G3145U, A3778G, and C6255U (Fig 2, S1 Table). Here again, each of these intersects one of the ARTIC primer binding sites (8_left, 11_left, 13_left and 20_right respectively, Fig 3). In aggregate, our set of lab-associated variants are significantly closer to ARTIC primer binding sites than would be expected by chance (P = 0.028, permutation test, Fig 3). Our results therefore suggest that variants intersecting or immediately surrounding commonly used primer binding sites should be subjected to particular scrutiny.

Another lab-associated variant, C22802G, also overlaps an ARTIC primer (76_left, S1 Table), but this is probably only a coincidence. The SARS-CoV-2 sequences carrying this variant were assembled from whole metatranscriptomic data without PCR selection. Instead, the cause appears to be misalignment of a human ribosomal RNA sequence that was incorporated into the consensuses for a subset of genomes produced by this group (Dr. Darrin Lemmer, *Pers*. *Comm*.). This highlights the broad range of possible causes of lab-associated variants.

It is more challenging to identify the specific sources of the other five lab-associated variants that we observed, but commonalities are informative. Three of these variants are associated with a single group and each is a G>U transversion (G3564U, G8790U, G24933U, S1 Table). Even more strikingly, each variant occurs in a GGU motif, suggesting a common molecular mechanism might underlie this set of lab-associated variants as well (*i*.*e*., GGU > GUU). Beyond these, G1149U and U153G are associated with two different sequencing groups, but do not show similar signatures as other variants (S1 Table). More generally, the fact that many apparently recurrent mutations are associated with genome sequences produced by individual lab groups suggests that consistent data processing or generation issues affect many sites. Sample contamination, which can be quite challenging to detect confidently, might also contribute to apparent mutational recurrence and might not strongly be lab-associated (S1 Text). However, we caution that this does not definitively prove that these apparent mutations are errors, but we believe it is prudent to remove these sites for most analyses until additional sequencing corroborates them.

### Lab-associated variants are consistent with simulated systematic error

**Fig 4:**
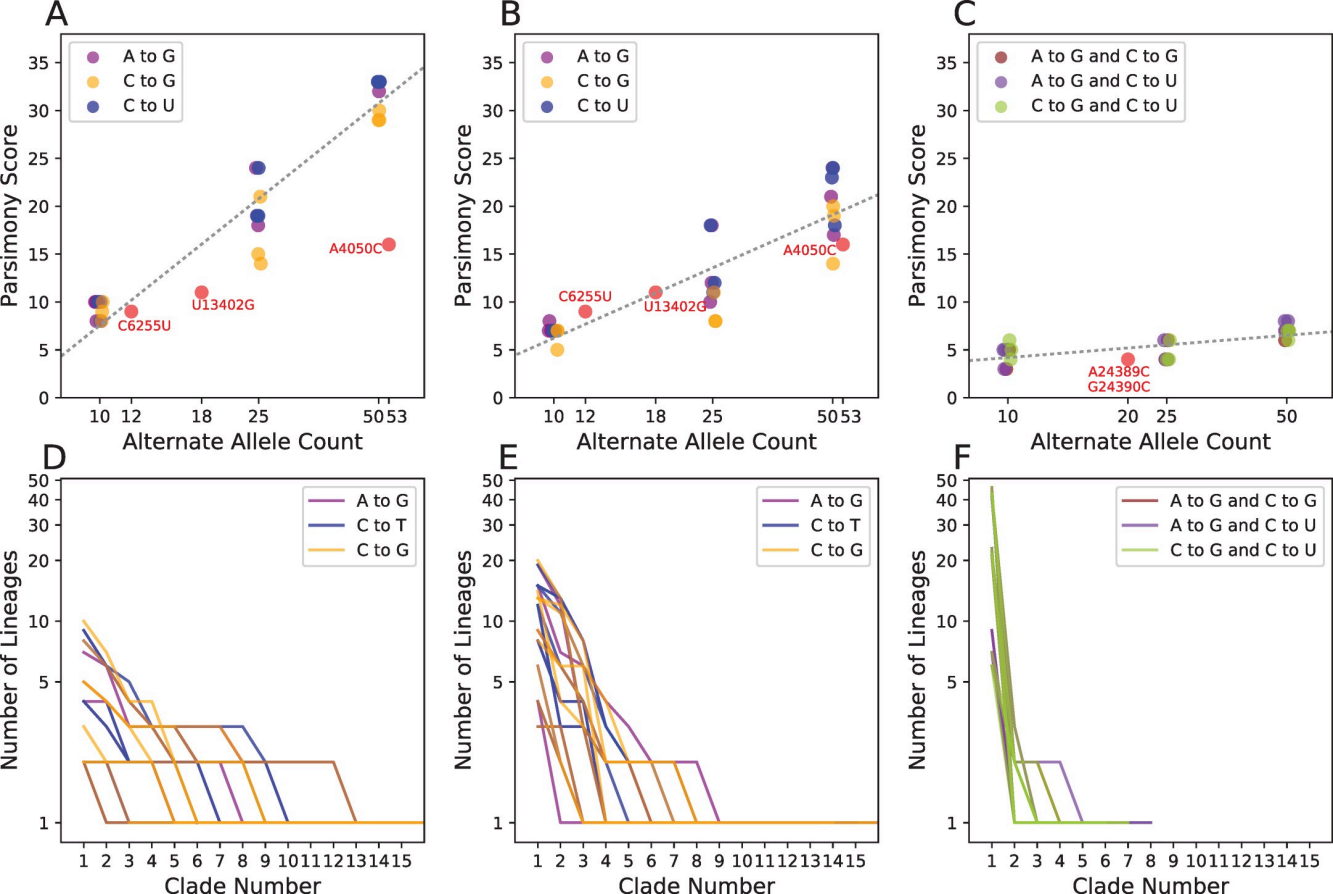
Parsimony scores at sites with introduced systematic errors. We added artificial errors to 10, 25, and 50 Australian (A) and early-March French (B) samples at the sites A11991G (purple), C22214G (blue), and C10029U (orange) in three replicates, then produced phylogenies and computed the parsimony score at each site. (C) We also introduced errors to the early-March French samples two at a time per sequence rather than individually. For comparison, we also show the values for three lab-associated variants (C6255U, U13402G, A4050C; A, B) and for pair of linked lab-associated variants (A24389C and G24390C; C). Each panel (A–C) contains a best-fit line (as in Fig 2A), for the relationship between log2 alternate allele count and parsimony in simulated error data (slopes = 10.0, 5.55, and 1.0). (D–F) Corresponding clade sizes arranged in descending order for error simulations in (A–C, respectively, as in Fig 2B).

To study how systematic errors affect phylogenetic inference and inferred properties of viral evolution, we experimentally introduced errors in replicate experiments. We found that the parsimony score displays a roughly linear relationship with the log of the alternate allele count, as it does for extremal sites in Nextstrain trees we examined built on different days in April, but with varying slope (Fig 4). This is expected because errors will sometimes occur in sample genomes whose positions are close on the real phylogeny and even in sister lineages. Tree-building methods could then group these samples into a single clade. Importantly, the effect of drawing samples together can cause systematic errors to appear heritable, and to reduce the apparent mutability of hypermutable sites.

Additionally, we find that viral genetic background and mutation type is an important contributor to this relationship. When errors are placed randomly across Australian samples (Fig 4A), we see much higher parsimony scores than when errors are placed only in samples from France collected between March 1 and March 17 (Fig 4B). The difference likely reflects the fact that the samples from France are more closely related. Because many of the lab-associated variants that we identified are derived from a similarly restricted time and geographic region as our samples from France, parsimony scores at those sites closely resemble these sets of simulated error (Fig 4B). More generally, this suggests genetic diversity in the viral population and in sampled isolates (which may be highly non-random relative to the population, *e*.*g*., if a single “transmission chain” is sampled) will affect detection of lab-associated variants. Importantly, the identification of lab-associated variants will become increasingly straightforward as the viral populations accumulate genetic diversity. We also observe that simulated errors associated with substitution events that are typically rare in SARS-CoV-2 evolution (*e*.*g*., C>G) have slightly lower parsimony scores. This is likely due to modeling nucleotide-specific mutation rates during tree-building where errors consistent with common substitutions (e.g., C>U) are less likely to be erroneously grouped. Importantly, our results suggest that a simple heuristic based on each position’s parsimony score and recurrence is sufficient to identify most lab-associated errors above very low frequencies. However, extremely infrequent lab-associated errors could remain challenging to identify.

Because systematic errors also affect the inferred tree, they can impact inferred patterns of mutational recurrence at other positions in the genome as well. In 50 out of 54 total experiments where we introduced a single recurrent error, we found that the parsimony score increased at other sites (range 2–44). This emphasizes the importance of identifying and excluding such variants prior to inferring the final tree and downstream analyses.

### Improvements and updates on detection of systematic errors, recurrent mutations, and other problematic sites

Due to the continuous increase in SARS-CoV-2 genome data availability, and because we sometimes improve our methods, we provide regular updates with up-to-date results and methodologies. Improvements in our methods and updated results are discussed at https://virological.org/t/issues-with-sars-cov-2-sequencing-data/473 while up-to-date recommendation for sites that we think need masking are available from https://github.com/W-L/ProblematicSites_SARS-CoV2/blob/master/problematic_sites_sarsCov2.vcf with metadata and format described at https://virological.org/t/masking-strategies-for-sars-cov-2-alignments/480. These forums are also helpful for us for receiving feedback from the scientific community.

Here, we list here a few of the key improvements to our methods. First, we now use information from ambiguous characters in genome sequences to better detect systematic errors. Ambiguity characters (in particular those other than “N”) are often used in consensus sequences when, at a certain genome position, more than one allele is detected above a frequency threshold within a sample. We often find these ambiguous characters at specific positions in sequences from particular labs. These highly ambiguous positions also tend to contain inferred systematic errors. For these reasons, frequent ambiguous characters can provide a reliable signal of biases in the sequencing process and can help detect lower frequency systematic errors. As another improvement, we now also account for the fact that in some cases the same putative systematic error is present in sequences from more than one sequencing lab, when different labs share the same sequencing protocols. We also now pay particular attention to variants that are linked, low-frequency, and nearby (<10bp distance) along the genome; these variants, if resulting from systematic errors, such as those at sites 24389 and 24390, can be very problematic as they can strongly skew phylogenetic inference and present a low parsimony score (see below). Finally, we study datasets of increased size as more genome sequences become available, and this gives us more power to detect systematic errors.

### Correlated lab-associated variants have large impacts on phylogenetic inference

If infrequent but highly correlated errors were introduced at different sites in many samples, this could cause more samples to be grouped into a clade than independent systematic errors. We might not easily detect these errors based on recurrence. Two lab-associated variants, A24389C and G24390C, are not just on adjacent genomic locations but are nearly perfectly correlated across samples. These sites have low parsimony scores when compared to other lab-associated variants (4 and 5, respectively, Fig 4C). When we introduced similar correlated errors, we found that the parsimony scores were lower than in single-error introduction experiments. Nonetheless, in only two error introduction experiments (out of 9) with 10 affected samples did we see a parsimony score as low as 3. Although low frequency and highly correlated error could be challenging to identify in general, we believe this is infrequent in our dataset (see S2 Text). In our most recent methods and analyses, we specifically tackle neighboring variants that are rare and linked (https://virological.org/t/issues-with-sars-cov-2-sequencing-data/473). A large proportion of these variants is likely the result of multi-nucleotide mutations; however, we also recommend caution when dealing with true mutation events with these characteristics, as they are typically not modeled in the phylogenetic context and can therefore cause biases when inferring branch lengths and the impacts of natural selection.

### Lab-associated variants affect phylogenetic inferences on scales relevant to local lineage tracing

**Fig 5:**
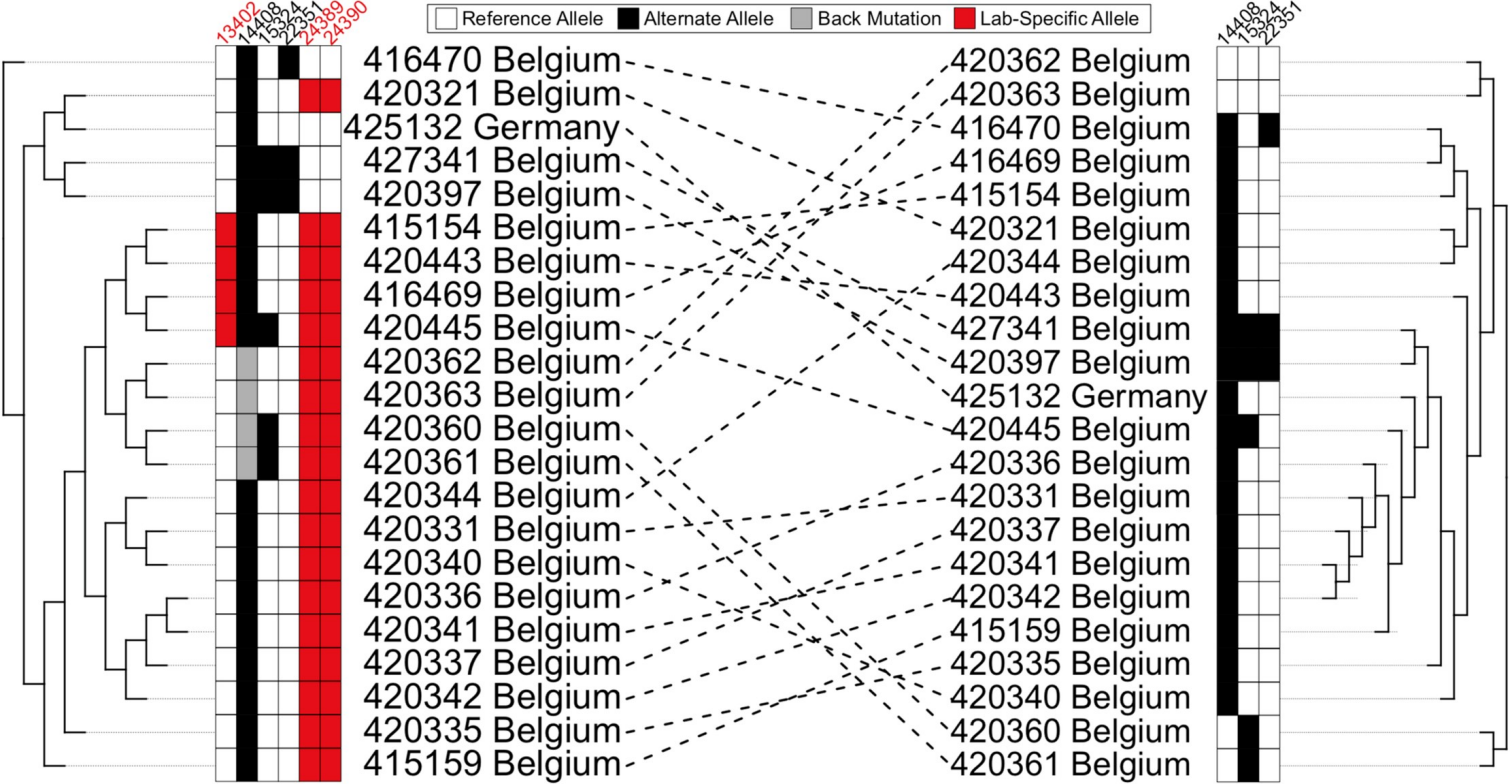
Lab-associated variants impact phylogenetic inferences. Part of the tree we obtained from the 4/19/2020 Nextstrain tree (left) compared to the corresponding part of tree after removal of sites with lab-associated variants (right). Lab-associated variants (red) can affect the inferred phylogeny and are associated with apparent back-mutation to the ancestral allele (grey in column 14408, left) at other sites (white). When lab-associated variants are removed, the resulting tree (right) shows no evidence for back-mutation at those sites (now white in column 14408), though several independent forward mutations remain evident.

To investigate the impacts of lab-specific variants on phylogenetic inference, we removed (“masked”) each of the 16 sites with a lab-associated variant (S1 Table) from the multiple sequence alignment. Importantly, removing lab-associated variants sometimes impacted phylogenetic patterns at other sites. For example, after removing all lab-associated variants, the evidence for back-mutations at C14408U is eliminated, while many forward-mutations remain (*e*.*g*., Fig 5). In fact, the overall parsimony score decreased by 16, increased for 54 sites and decreased for 53 sites on the tree that we inferred after removing all of the lab-associated variants relative to the tree inferred including all sites. Additionally, we find that many samples containing lab-associated variants have been repositioned on local topologies (*e*.*g*., Fig 5). Furthermore, in some cases the placement of closely related lineages that are unaffected by lab-associated variants is also affected (S2 Fig). These variants therefore affect phylogenetic inferences at scales relevant to local lineage tracing, which may obscure dynamics of local transmission.

**Fig 6:**
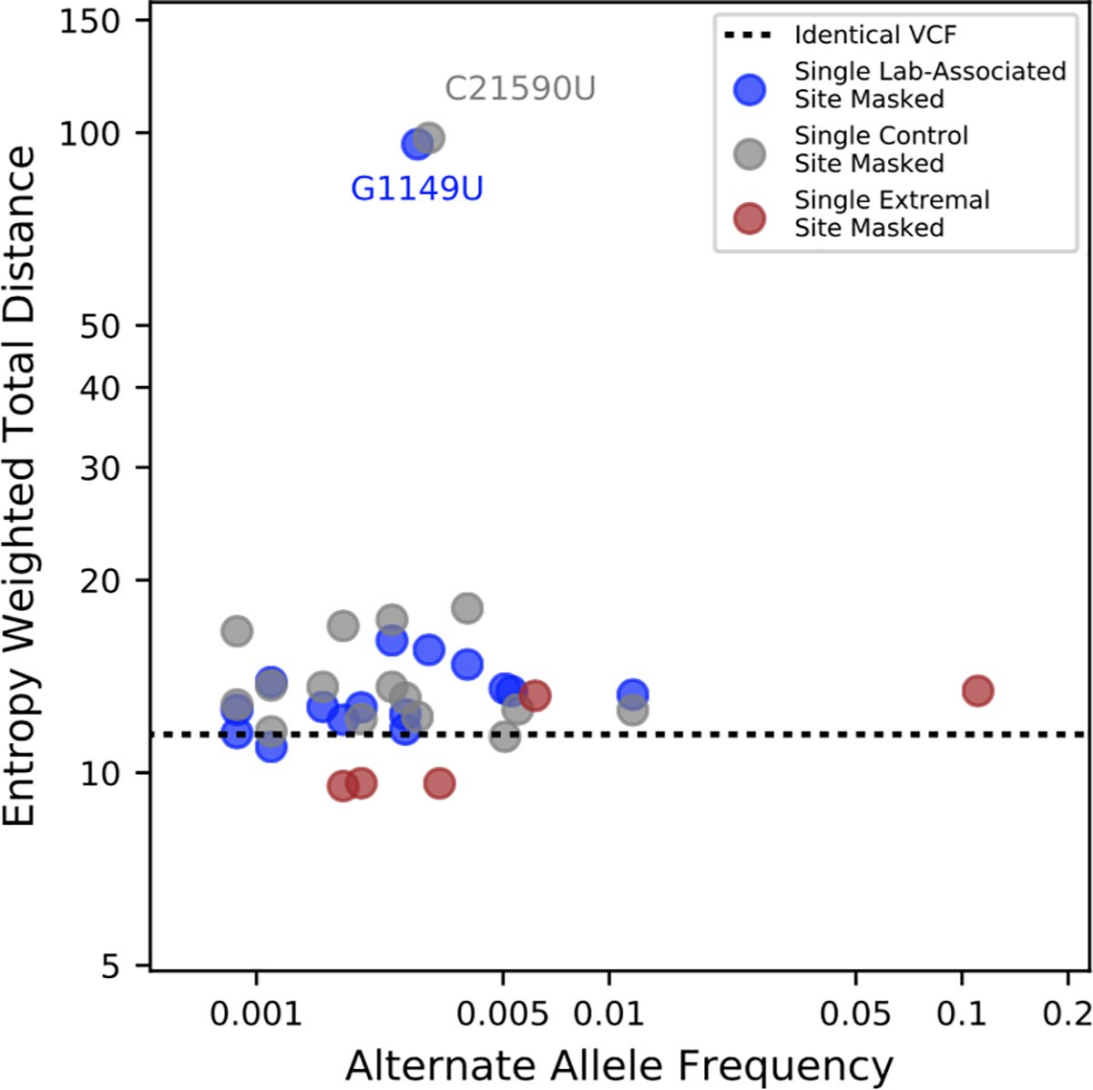
The relationship between alternate allele frequencies of lab-associated variants and effect of masking on inferred tree topology. Entropy-weighted total distances relative to the reference maximum likelihood phylogeny are shown for phylogenies constructed after masking individual sites. Blue points correspond to sites with lab-specific alternate alleles, grey points correspond to control sites with parsimony scores of 1 and similar alternate allele frequencies to the sites with lab-specific alternate alleles, and brown points correspond to non-lab-specific extremal sites. The black horizontal line indicates the entropy-weighted total distance value for a maximum likelihood phylogeny constructed from an alignment identical to that of the reference phylogeny. Two outliers, C21590U (control) and G1149U (lab-associated), have outsize effects on inferred tree topology.

To examine the effect of each lab-associated variant and the other extremal sites in isolation from one another, we individually masked each site and inferred a phylogeny. As a comparison, we also masked a set of sites that have similar alternate allele frequencies as the lab-associated variants, but each has a parsimony score of one. The distributions of entropy-weighted total distance (a measure of distance between trees, described below) are remarkably similar when masking individual lab-associated sites, other extremal sites, and our control sites (Fig 6). Most exceed the distance we observed when we independently inferred two trees from the same input alignment (dashed black line). Our results therefore suggest that the impact on tree-building of the lab-associated and extremal sites we identified can be on a par with that of real mutations, although the effects are typically small on the scale of whole topologies, as expected given their typically low allele frequencies (Fig 6, S3 Fig).

Phylogenies made after removing two variants, one control and one lab-associated, are outliers for entropy-weighted total distance (Fig 6, S4 Fig) and other tree distance statistics (S3 Fig). In each case, however, the likelihood of the tree produced from the full dataset is actually higher (S5 Table), suggesting that our tree-building method discovered a different locally optimal but less favorable topology rather than a dramatic impact of each site individually. These results suggest higher level uncertainty in the tree topology largely independent of the effects of lab-associated variants.

### Recurrent mutations not associated with a lab reflect the mutation spectrum of the SARS-CoV-2 genome

**Fig 7:**
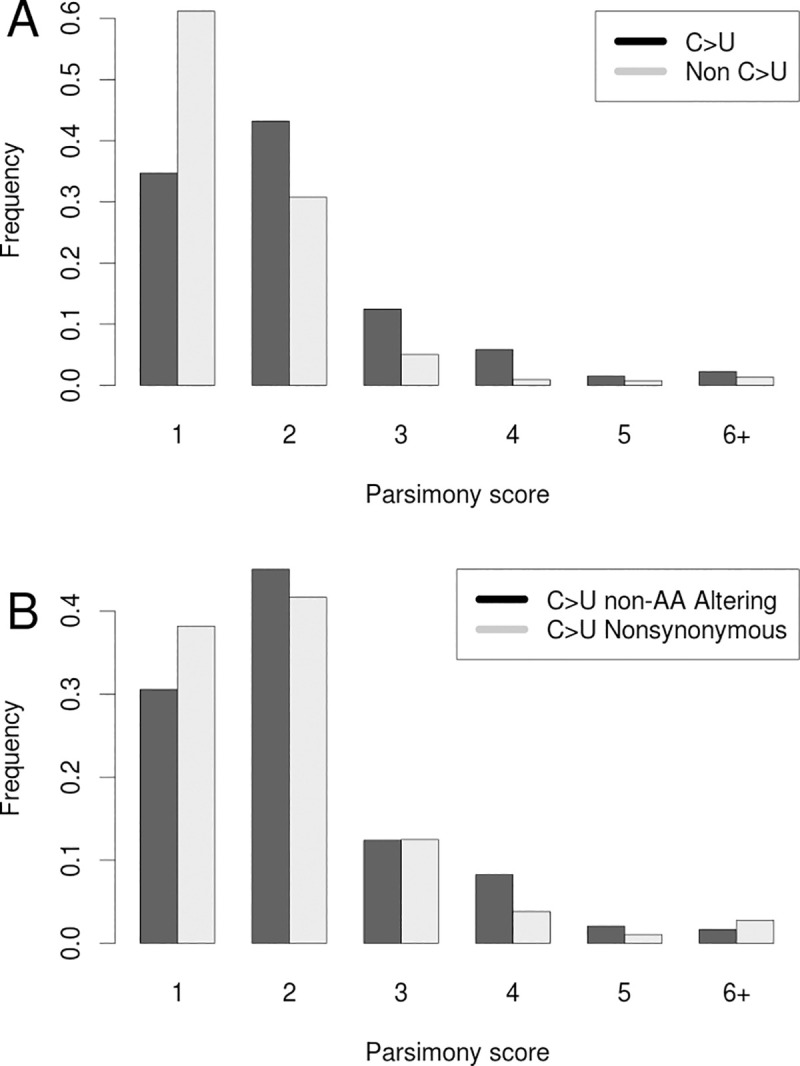
Recurrence of mutations during SARS-CoV-2 evolution. (A) Frequencies of parsimony scores for C>U (Black) vs all other mutation types (Grey). (B) Frequencies of parsimony scores for C>U mutations that do affect amino acid sequences (non-synonymous; Grey), and those that do not affect amino acid sequences (synonymous; Black).

Hypermutation rather than positive selection may explain many remaining highly recurrent sites. Previous analyses have indicated that the number of C>U mutations is exceptionally high relative to other mutation types in the viral genome [11,26,45,49]. This class of mutations should show increased evidence of recurring multiple times because they experience elevated mutation rates [26]. Indeed, parsimony scores at sites containing C>U mutations are significantly higher than those for all other mutation types (P < 2.2e-16, Wilcoxon Test, Fig 7). Furthermore, parsimony scores at C>U sites also significantly exceed those at G>A (P < 6e-12) as well as U>C (P ≈ 1e-10) sites. This mutational bias might be driven by APOBEC editing of the viral genome [26,45,46,49–51]. Consistent with previous results [45,46,49–51], we find that 5’-[U|A]C>U mutation occurs more frequently than 5’-[C|G]C>U (P = 0.0501), but we do not see a similar effect for 3’ flanking sites at 5’-C>U[U|A] relative to 5’-C>U[G|C] mutations (P = 0.378). The highly biased spectrum of C>U mutations and the correlation with local sequence context implies that the plus-stranded virus biology may be leading to recurrent C>U mutations [49].

Of the 83 highly recurrent mutations with parsimony score greater than 3, 50 are bi-allelic, not strongly lab-associated, and have an alternate allele frequency less than 0.01. Of these, 42 are C>U mutations. This is a significant excess of C>U mutations relative to the rate among non-singleton bi-allelic sites with parsimony score ≼3 (P = 3.658e-07, Fisher’s exact test). Additionally, synonymous C>U mutations (those that do not affect the underlying amino acid sequences) display higher parsimony scores than do non-synonymous C>U mutations (that do affect amino acid sequences; P = 0.0553, Wilcoxon test, Fig 7). This suggests that negative selection has played a role in shaping the distribution of highly recurrent mutations by purging strongly deleterious alleles.

Evidence suggests that any contribution of sequencing error to the excess of C>U mutation is small. Alternate alleles at 81.4% of sites with parsimony >3 are corroborated by more than one sequencing technology. Of those, 77% of bi-allelic sites are C>U transitions (S2 Table). Illumina C>T errors in raw cDNA-derived sequencing reads are typically enriched in the contexts of flanking G regions [52,53], but here we do not see this pattern. Similarly, nanopore sequencing typically creates errors in homopolymer stretches [54], but we only see a few recurrent mutations associated with such regions, notably the extremal sites C11074U, and C21575U, which abut poly-U stretches in the genome and might result from replication slippage (see also G11083U). Despite this intriguing observation, a recent analysis formally tested for and did not find enrichment of homoplastic evolution at positions adjacent to or within homopolymer stretches [26] and we therefore do not recommend uniformly masking positions adjacent to homopolymer sequences. It is possible that the excess of C>U mutations is driven in part by high error rates during reverse transcription [55–58], which is required for cDNA sequencing. However, C>U mutation is overrepresented in high-frequency mutations as well (9/20 frequency > 0.025 mutations are C>U, S3 Table), indicating that this bias likely reflects a true mutational process. Additionally, these mutations are approximately as distant from ARTIC primer binding sites as would be expected by chance (P = 0.7851, Permutation Test, S2 Table). Collectively, our results suggest that neither library preparation nor sequencing error is the major driving force behind biased C>U mutation observed at highly recurrent mutations that are not strongly associated with a single lab. However, even if real, the existence of these highly recurrent mutations does not require that they are inherited (it must be that many viral mutations are never transmitted), in which case they will occur on terminal branches in the real tree and their phylogenetic behavior should be the same as systematic errors.

### Possible mitigations for lab-associated variants and highly recurrent mutations

We have proposed a simple heuristic approach to detect lab-associated variants, summarized as first identifying sites that apparently experience mutations on at least four independent branches of the SARS-CoV-2 tree, and then extracting the set where 80% or more of the alternate allele comes from sequences produced by a single lab. These are classified as lab-associated variants. Then for all sites we plot parsimony score versus log2 of alternate allele count and determine a set of extremal sites as described in Methods. We recommend that lab-associated and most extremal variants be masked for the purposes of constructing a phylogenetic tree to be used in downstream analyses. One exception here is extremal site 11083, which is sufficiently high frequency that it affects inference of the deepest branches of the tree. We suggest that it should be included during phylogenetic inference. However, alternative masking strategies that remove small clades containing apparent forward and backward mutations at high-frequency sites might also be effective and will be investigated going forward. Many downstream analyses following tree-building should consider masking 11083 as well. After masking the set of lab-associated and extremal sites, the samples which previously contained them can be retained in phylogenetic inference and downstream analyses. Tracks identifying these sites are available on the UCSC Genome Browser and in S1 Table.

Though not a focus here, we emphasize that filtering for genomic regions that are difficult to assemble or align (*e*.*g*., those used by Nextstrain to filter the ends of chromosomes as defined here https://github.com/nextstrain/ncov) should also be rigorously employed. In fact, in light of our discovery of a possible lab-associated variant at position 153, which is just within the usual filtering range, we suggest that it may be preferable to simply mask the full 5’ and 3’ UTR regions, which are typically harder to assemble and align confidently.

**Fig 8:**
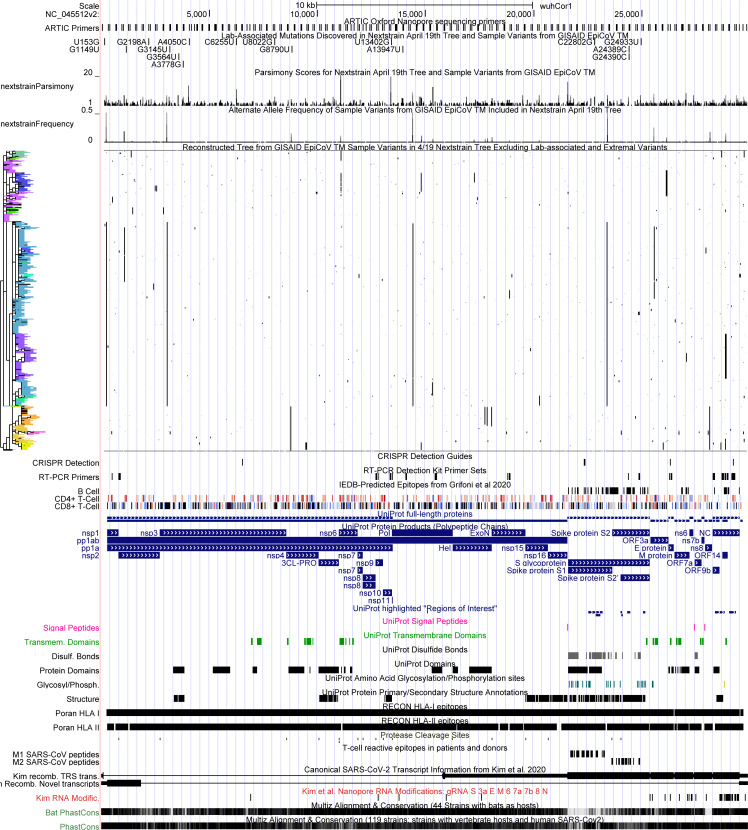
UCSC Genome Browser view of all lab-associated variants in the context of parsimony scores, alternate allele frequencies, the full genetic variation dataset with phylogenetic tree constructed after removing lab-associated and extremal variants. This genetic variation data can be cross-referenced against many other diverse datasets available in the UCSC SARS-CoV-2 Genome Browser. Interactive view: http://genome.ucsc.edu/s/SARS_CoV2/labAssocMutsAll.

To examine the aggregate effect of lab-associated and extremal variants, we inferred a tree from the full dataset (“total tree”), and another after masking all lab-associated and extremal variants except 11083 (“filtered tree”) using IQ-TREE 2 with 1000 ultrafast bootstraps [59,60]. We then collapsed all branches that do not contain a mutation into a polytomy. In contrast to the single site masking experiments above, here the topologies of the two maximum likelihood consensus trees differ significantly. The symmetric entropy-weighted total distance between the two topologies is not large, 9.4, but the fit of the filtered tree to the masked multiple alignment improved by 189 log-likelihood units relative to the total tree. IQ-TREE 2 is stochastic and each run may not yield an optimal topology. We therefore confirmed that the filtered tree is a better fit for the filtered data by producing 10 replicate trees using the total and filtered datasets. We found that the likelihood of the filtered trees was highest for the filtered datasets for nine of the ten replicated filtered trees. The final replicate topology had a much lower likelihood suggesting that IQ Tree inferred a suboptimal topology in that case. Collectively our results indicate that the inferred topology is indeed affected by excluding lab-associated and extremal variants. Below, we show that confident relationships at higher branches in the topology are minimally affected relative to other widely-used phylogenies, which were inferred including lab-associated and extremal variants. Our phylogeny produced following these masking recommendations is available from the UCSC Genome Browser (Fig 8).

Many of the most intriguing and evolutionarily relevant biological phenomena, such as viral recombination and recurrent mutation, explicitly require inferences based on homoplastic mutations. Special caution is clearly warranted. For these analyses, it is still necessary to mask lab-associated variants and extremal sites because they can destabilize phylogenetic inference, but clearly one could not exclude all homoplasies. In light of significant phylogenetic uncertainty, which we address below, we recommend that each analysis be repeated across alternative possible tree topologies to confirm the robustness of biological inferences. However, this is not without significant challenges and the most general solution for confirming recurrent mutation or recombination is heritability. If a mutant or recombinant lineage grows sufficiently large and is corroborated by many labs, we can be much more confident [27]. We therefore suggest that evidence of heritability and independent sequence confirmation should be required to support inferences of either recurrent mutation or recombination.

Although our results above indicate that our approach has worked well on these SARS-COV-2 datasets, we caution that the identification of systematic errors based on the apparent recurrence of mutations carries important challenges. In particular, this approach implicitly assumes that the real signal in a set of samples is sufficient to infer a reasonably correct global phylogeny despite the noise induced by the presence of systematic errors. If systematic errors are frequent relative to true mutations or highly correlated, this might impact the initial phylogeny to the point that they could not be detected. By removing the most obviously discordant sites, one could “lock in” errant variants and thereby reinforce the incorrect phylogeny. A second related consideration is that systematic errors could be correlated with real genetic variation (*e*.*g*., if mutations present in a primer binding site affect the resulting consensus sequences). Because these errors would likely be strongly correlated with the real phylogeny, their detection based on apparent homoplasy could be difficult.

### Exploring data quality and mutational recurrence using our tools

SARS-CoV-2 sequence data is growing at an incredible pace. Here we developed tools to enable investigations of similar patterns in updated and additional datasets. To summarize: (1) we provide a method for rapidly computing parsimony scores to identify highly recurrent positions; (2) we provide an approach for identification of unusually recurrent sites relative to their allele frequencies (here, termed extremal); (3) we provide an approach for semi-automated metadata correction (See Methods), which improved detection of lab-associated variants; and (4) we provide a method for identifying the set of highly homoplastic variants that are strongly associated with individual sequencing labs. Our heuristic cutoffs appear to perform well in the datasets we examined, but the program is designed to empower users to explore other datasets and other filters as well. Software to perform each analysis are provided via GitHub (https://github.com/lgozasht/COVID-19-Lab-Specific-Bias-Filter and https://github.com/yatisht/strain_phylogenetics). Further, (5) we provide a regularly updated list in VCF format (Variant Call Format, [61]]) of sites that we recommend masking based on our analyses, available from https://github.com/W-L/ProblematicSites_SARS-CoV2/blob/master/problematic_sites_sarsCov2.vcf and explained at https://virological.org/t/masking-strategies-for-sars-cov-2-alignments/480; and (6) we regularly update and discuss our methods on virological.org https://virological.org/t/issues-with-sars-cov-2-sequencing-data/473 and welcome feedback from the scientific community.

### Visualizing data quality, genetic variation and correlation via the UCSC SARS-CoV-2 Genome Browser

Data visualization remains one of the most powerful mechanisms for identifying unusual patterns and possible errors in genome sequence data (*e*.*g*., Fig 3, above). Therefore, as an integral part of this work, we provide powerful data exploration and visualization tools that can be applied to future variation datasets as well. Output from our programs for computing parsimony scores and detecting lab-association variants can be imported directly into the UCSC SARS-CoV-2 Genome Browser [62] as custom tracks to facilitate visual exploration of suspect mutations with a user-defined VCF file and tree. This is a very useful visualization framework for data quality control and for investigating the root causes of highly recurrent mutation. For example, it is straightforward to explore the relationships between phylogeny, genetic variation, and functional genomic annotations (Fig 8).

Researchers can upload their own aligned SARS-CoV-2 genome samples and phylogenetic trees to the SARS-CoV-2 Genome Browser in order to compare their phylogenetic analyses to those from Nextstrain and COG-UK, and also to look at the specific molecular features of the clades that their phylogenetic analysis identifies (S4 Text). These molecular features include widely used primer pairs (as in Fig 3) as well as CRISPR guides, predicted and validated epitopes for CD4+ and CD8+ T-cells, key functional sites on the viral genome including cleavage sites for viral proteases PL-PRO and 3CL-PRO as well as cleavage sites for host proteases, locations of important RNA secondary structures, the locations of the transcriptional regulatory sequences, locations of protein phosphorylation and glycosylation sites, identification of sites in the virus that are highly conserved or rapidly evolving in closely related viruses in bats and other mammals, as well as a lively “crowd-sourced annotation” set where any researcher can point out additional sites on the viral genome of special functional, diagnostic, or therapeutic significance [62]. This helps researchers to quickly determine if alternate alleles they believe characterize a new viral clade may be significant beyond their role as epidemiological markers. Instructions for producing custom genome-browser tracks for a given phylogeny and variation dataset are provided in S4 Text.

### Phylogenetic uncertainty and facilitating tree comparisons across analyses

In the second part of this work, we address concerns arising from phylogenetic uncertainty. As expected for a relatively slowly evolving and rapidly expanding viral population [63], there is substantial uncertainty in the SARS-CoV-2 phylogeny. This extends well beyond the typically localized impacts of lab-associated and highly recurrent variants, and instead derives from the fact that most branches in the SARS-CoV-2 phylogeny are supported by few mutations. Undoubtedly, thousands of unique phylogenies will be produced by groups studying this viral outbreak, and these may sometimes support conflicting evolutionary relationships. We therefore sought to provide tools to facilitate interpretation of commonalities and differences among such large phylogenies.

### A tree comparison algorithm using entropy-weighted matching splits

There are many metrics for measuring the total distance between two or more phylogenetic trees [64–69]. One popular metric (Maximum Cluster distance (MCdist)) also identifies the best-matching clades between the two trees. A clade in a rooted tree splits the leaf nodes of that tree into two sets: those inside the clade and those outside the clade. Given a clade C in tree T and a clade C’ in tree T’, the split distance between C and C’ is the number of leaves that have to be moved so that the split for C in T becomes equal to the split for C’ in T’. The (nonsymmetric) correspondence between the clades of T and the clades of T’ established by minimizing the split distance is referred to as the “maximum cluster alignment” or “best split alignment” from T to T’, [64]. This is particularly appealing here because we aim to facilitate comparisons across phylogenetic trees both globally and at individual clades.

**Fig 9:**
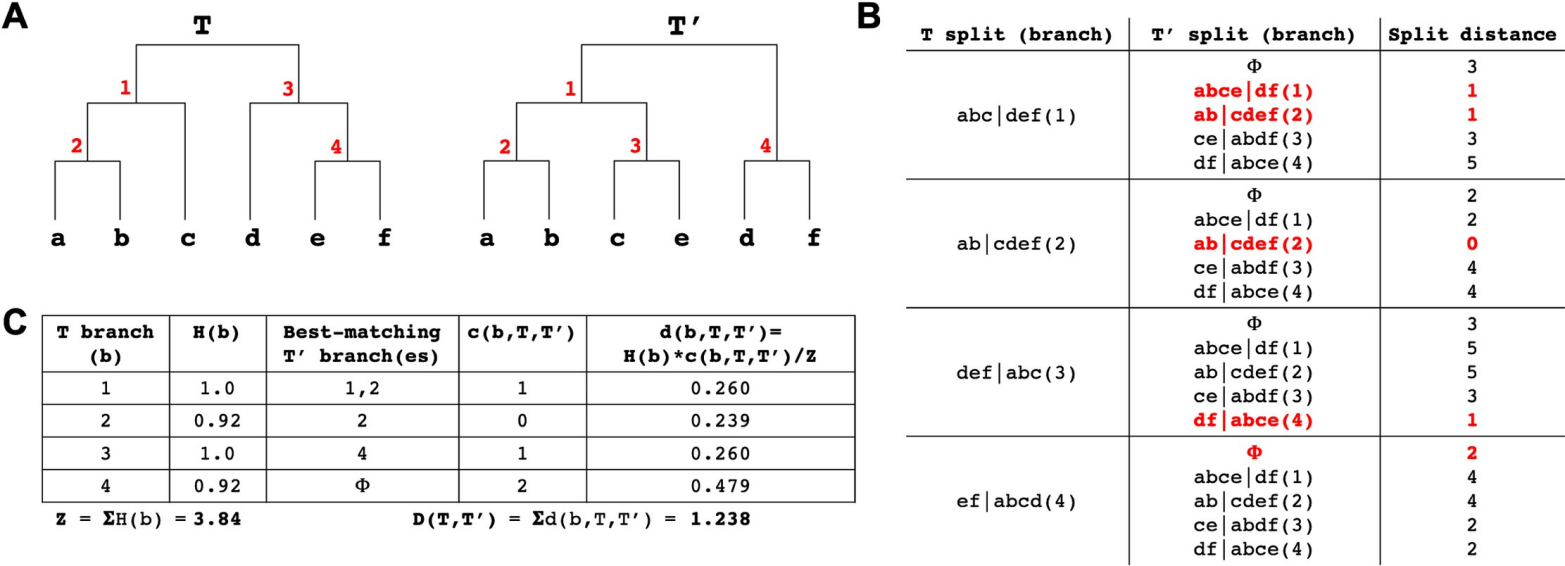
Entropy-weighted distance statistic. (A) Example trees (T and T’) for this comparison with identical sets of leaves but different topologies. Internal branches are labelled in red. (B) The split distance statistic for each T node (see Methods for notation). Split distance of each T split (branch) from all T’ splits plus a “garbage node” (ɸ) containing a null set of leaves, with the matching split distance and its corresponding T’ split (branch) for each T split (branch) highlighted in red. Multiple T’ splits can match a T split but the garbage node is given precedence (as is the case in T branch 4). (C) Table showing the entropy, best-matching T’ branch(es), matching split distance and entropy-weighted matching split distance for each branch in T, as well as the entropy-weighted total distance D(T,T’) between T and T’.

We implemented a modified version of MCdist in https://github.com/yatisht/strain_phylogenetics to compare two trees, T and T’, restricted to the same set of samples, with two improvements. First, we proportionally weighted the split distance between each clade C of T to the best matching clade in T’ by the entropy of C, i.e., by H(p) = -p log2(p)—(1-p) log2(1-p) where p is the fraction of leaves from T that are in C (see Methods, Fig 9). The entropy-weighted matching split distance emphasizes the importance of the clades in T in terms of how much information about the leaves they carry, which helps highlight clades where the most dramatic changes have occurred. The sum, over all clades in T, of the entropy-weighted matching split distance to the best-matching clade in T’ is referred to as entropy-weighted total distance from T to T’. Second, we label all internal branches in T and T’, and identify the most similar branches in both trees based on the clades they define. When multiple branches in T’ match the branch b in T with the same best split distance, we report all best-matching branches (Methods). Additionally, we confirmed that our statistic is a robust measure of tree distance, judging by the strong correlation with other frequently used tree distance metrics (S3 Text). Our implementation can compute this statistic for two trees of size 10,000 leaves in just 20 minutes on a single CPU, so it scales to the large trees required for SARS-CoV-2 phylogenetics.

### A fast algorithm for producing tanglegrams for trees with thousands of leaves

A tanglegram is the most often used method of visualizing the topological difference between two rooted phylogenetic trees defined on the same set of leaf taxa (here, termed samples)[70]. We expect that tanglegrams will have a wide use for analyzing and comparing different SARS-CoV-2 phylogenies. Tanglegrams plot two trees side-by-side with their common leaves connected by straight lines (*e*.*g*., S5 Fig). For visually appealing and informative tanglegrams, clades in both trees are arranged in a similar vertical order (given the tree topology constraints) with minimum crossing of connecting lines with each other. While there are a number of tree node “rotation” algorithms that optimize tanglegrams for visual appeal [70,71], we found none of the available implementations that we tested [71,72] worked reasonably for phylogenies as large as SARS-CoV-2 phylogenies, either producing unacceptable results or not able to finish the computation. We therefore developed a fast heuristic approach that produces vastly improved tanglegrams (Methods, S5 Fig, https://github.com/yatisht/strain_phylogenetics). Our approach takes approximately one minute for the tanglegrams we show here, and we use this heuristic for displaying tanglegrams throughout the text.

### Nextstrain phylogenies vary significantly over time

**Fig 10:**
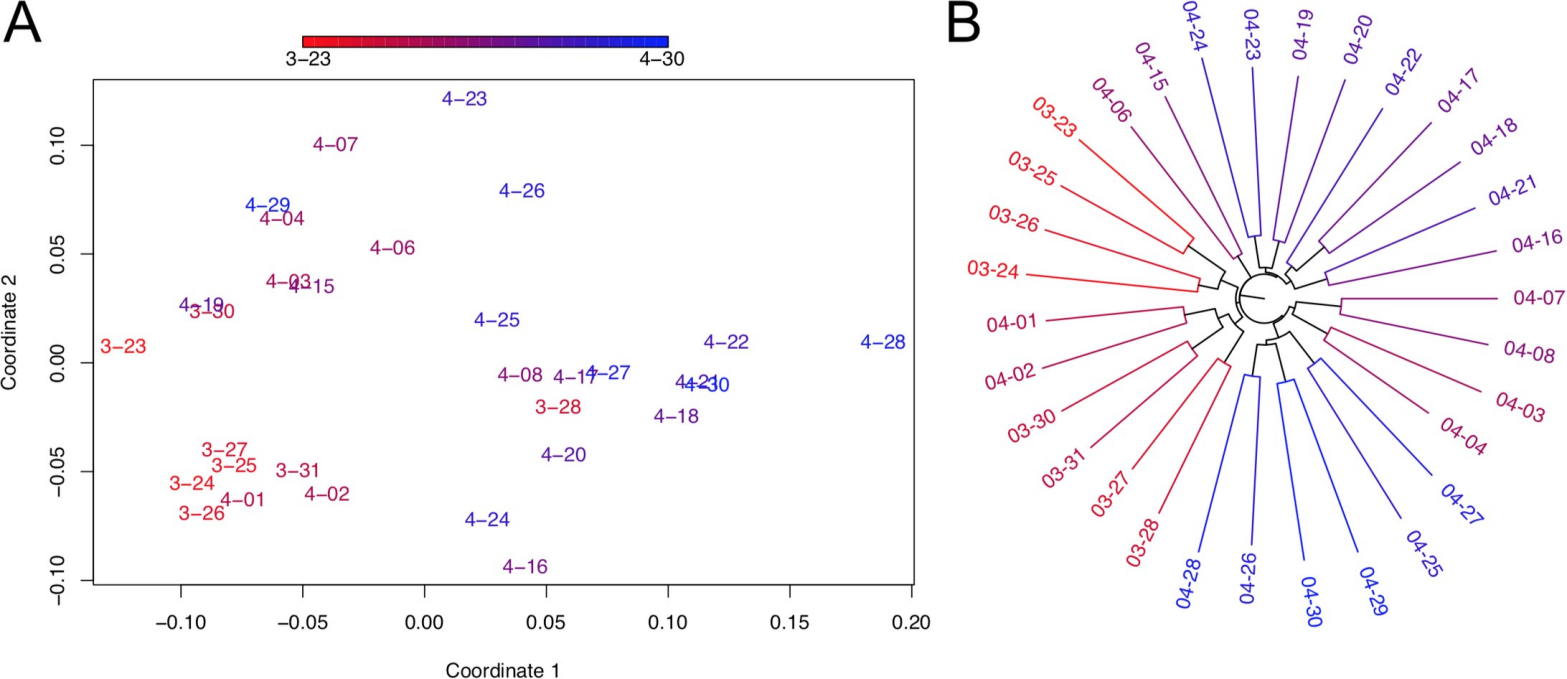
Comparisons of Nextstrain trees over time. (A) Multidimensional scaling of normalized entropy-weighted total distances among phylogenetic trees produced by Nextstrain from March and April. Each topology is labelled with its date and dates are depicted in a color gradient from 3/23 (red) to 4/30 (blue). Coordinates 1 and 2 are plotted here and each contributes 34% and 15% of the total variance explained, respectively. (B) Relationships between Nextstrain phylogenies are shown in a tree-of-trees, “meta-tree” [67] we constructed, which displays the distances among topologies of the constitutive trees.

We next explored differences among trees made by the same group from slightly different sample sets with the goal of understanding phylogenetic stability as new samples are incorporated. For comparison, we restricted 31 Nextstrain trees produced between March 23, 2020 and April 30, 2020 to just the 468 samples they all have in common. Comparing topologies, we found that a number of these 468 samples moved back and forth between different clade designations during the month (S5 Fig), including samples in the specific clades (A1a, A2, A2a, A6, A7, B, B1, B2, B4) named and analyzed by the Nexstrain consortium during this period (e.g., S6 Table). Note that the Nextstrain clade ID system was updated while we were finalizing this work [73]. We then measured all pairwise tree distances between restricted trees and found that they varied widely (normalized entropy-weighted total distances ranged from 0.089 to 0.352, Fig 10). There is therefore substantial variation in Nextstrain phylogenies over time.

Multidimensional scaling (MDS) of the pairwise distances among each topology, as well as meta-tree analysis [67] reveals a strong relationship between topologies and the date that each tree was produced (Fig 10). In particular, the first MDS coordinate is strongly correlated with the release date of the tree (Spearman’s rho = 0.688, P = 3.087e-05). This effect is expected and likely driven, at least in part, by the impact of the sample set used to produce the resulting tree, which necessarily changes as new data are incorporated. Indeed, the proportion of overlapping samples used in constructing each pair of trees is strongly negatively correlated with the normalized entropy-weighted total distance between their topologies (r = -0.384, P = 4e-05, Mantel test), while the set of 468 samples for which we analyze topology is held fixed for all trees. These tools provide the research community a method for tracking the phylogenies of SARS-CoV-2 as the pandemic progresses and phylogenies are produced for larger and larger sample sets. The tools can detect when older clades are confirmed as new samples accumulate, stabilizing inference of these clades, as well as track new subclades as they grow. If inconsistent data are causing persistent clade instability, which may result from lab-associated sequencing errors or actual recombination, it should be visible in this analysis.

### Higher-level branches are remarkably consistent across analyses

Even if it was possible to obtain error-free data and multiple alignments as well as have all groups use those same data, different tree inference approaches can produce different topologies. Furthermore, there is substantial uncertainty inherent to SARS-CoV-2 evolution because there are few mutations that uniquely mark each branch. Nonetheless, it is essential that epidemiologists studying the pandemic be able to communicate phylogenetically informed observations [18,43]. As discussed above, the clade placements of individual samples, even when inferred by the same group, can vary as different datasets are incorporated into the tree construction process (*e*.*g*., S4 Table, Fig 10). Differences between groups are expected to be even more pronounced. This threatens to leave the community with a communication problem in clade characterization and naming from various different phylogenetic trees. Indeed, the names used for Nextstrain’s original clades (A1a, A2, A2a, A6, A7, B, B1, B2, B4) bear no relationship to the lineage names (A, B, A.1, A.2, B.1, B.2, A.1.1, etc.) suggested by the COG-UK consortium [18,19], and without a 1–1 correspondence between the topologically defined clades in their respective phylogenetic trees, it is difficult to translate nomenclature in order to conduct precise scientific discourse pertaining to the evolutionary conclusions reached by these groups. Adding further difficulty to this situation, clade naming approaches based on phylogenies must themselves be subject to change as the pandemic spreads and as the evolution of new genotypes requires naming new clades and modifying existing clades. As clade based comparisons are an essential part of consistent scientific discourse, tools are needed to ameliorate these difficulties.

**Fig 11:**
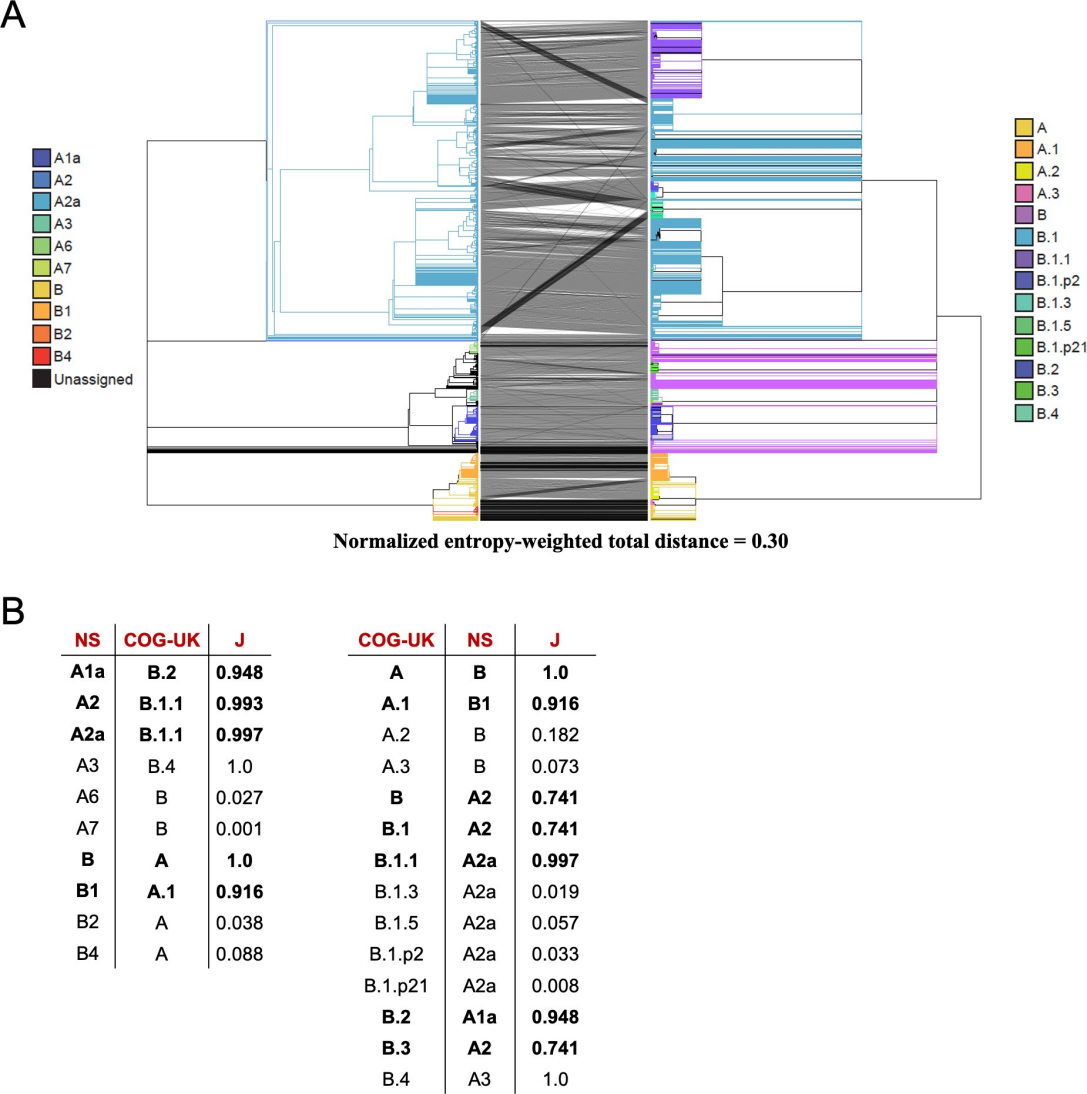
Comparison of Nextstrain and COG-UK trees. (A) A tanglegram of the Nextstrain tree from 4/19 (left) with the COG-UK tree from 4/24 (right). Each tree has 4167 samples. (B) The COG-UK clades (which they term “lineages”) having the highest Jaccard similarity coefficient (J) with each Nextstrain (NS) named clade and vice versa, where the Jaccard similarity coefficient is computed using the set of samples from the root of that clade. Clades with more than 200 samples are shown in bold font and called “big”, the others “small”. While the naming schemes differ, for each big Nexstrain clade there is a closely corresponding COG-UK clade, and vice-versa.

To explore the differences among available phylogenies and to provide guidelines for clade-based comparisons across possible evolutionary histories, we used our approach to identify the correspondence between the Nextstrain phylogeny produced on April 19, 2020 and the COG-UK phylogeny produced on April 24, 2020 (Fig 11A). We observe good agreement between the big Nextstrain named clades and their corresponding best matching named clades in the COG-UK tree and vice versa (e.g., “A2a” clade in Nextstrain, “B.1” clade in COG-UK, etc., Fig 11B), suggesting that these clades are reasonably stable across different analyses. However, in small named subclades within those prominent clades, there are many noteworthy differences between the two topologies, and the overall congruence is significantly reduced (Fig 11A). In addition to differences in methodology, this reflects a difference in the time when clades were originally named and the intent of each nomenclature system. Nextstrain named clades much earlier and many did not increase in size subsequently; others have since emerged and were named by COG-UK later. Additionally, the COG-UK system is intentionally dynamic and clades that have become inactive are removed. As a consequence, some clades do not have an obvious named analog in the two systems, resulting in low similarities (Fig 11B).

Perhaps the most obvious difference between the topologies is that the COG-UK tree has many more large polytomies (Fig 11A). This reflects the decisions motivating their analysis [18,74], where the authors’ goal is to provide a well-supported and stable topology to facilitate lucid communication about viral lineages for evolutionary as well as epidemiological studies. This contrasts with the Nextstrain project’s primary goal of up-to-date transmission tracing. As is typical in phylogenetics, topological stability comes as a tradeoff against the cost of articulation in the branches. Because of the many different motivations for constructing phylogenetic trees, it is a certainty that many independent trees will be used to study the evolution of SARS-CoV-2. Comparisons using our approaches can enable communication about evolving viral lineages across disparate analyses by facilitating the identification and visualization of the most closely matching clades.

### Higher branches in our tree closely mirror a Nextstrain “consensus” tree and the COG-UK Tree

**Fig 12:**
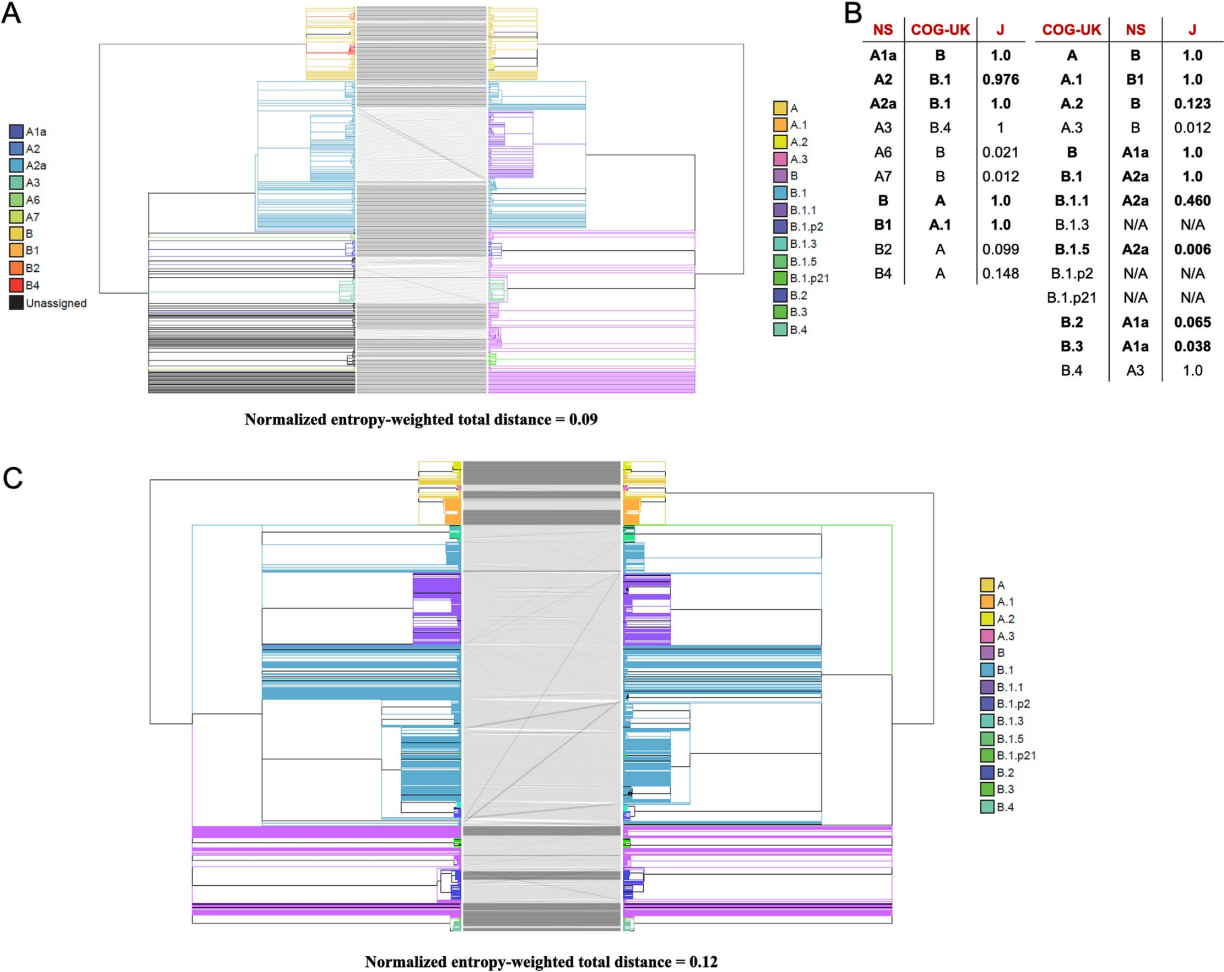
Comparison of Nextstrain and the COG-UK trees. (A) A tanglegram of our Nextstrain consensus tree (left) and COG-UK tree from 4/24 (right). Each tree has 422 samples. (B) The COG-UK lineages having the highest Jaccard similarity coefficient (J) with each Nextstrain consensus (NS) named clade and vice versa. Big clades defined in Fig 11 (those containing 200 or more samples in the Fig 11A trees) are in bold. Lineages in ‘N/A’ (B.1.3, B.1.p2 and B.1.p21) were pruned out as a result of restricting the trees to common samples. (C) A tanglegram of our tree produced after masking all lab-associated and extremal variants except 11083 (left) and COG-UK tree from 4/24 (right). Each tree has 4172 samples and the samples (branches) have been colored based on COG-UK lineage labels.

To identify stable nodes across analyses we compared a Nextstrain “consensus tree” and the COG-UK tree. To do this, we produced a majority rule clade consensus tree [75] for the 422 common samples in 31 trees produced by Nextstrain between 3/23 to 4/30, and restricted the COG-UK tree to these same samples. We find exceptionally good congruence between our Nextstrain consensus and the COG-UK phylogenies (Fig 12A), even though the inference methods differed substantially. Specifically, the COG-UK tree is built using a more typical bootstrapping approach [60], whereas our approach for building a Nextstrain “consensus” from trees produced on subsequent days would resemble a kind of “bootstrapping by samples” approach. This congruence reaffirms the idea that the COG-UK tree provides a stable “backbone” to enable direct conversations in epidemiology. Nonetheless, we still observe several small rearrangements between the two topologies, suggesting that both will likely be subject to clade refinements in the future.

We also observed good overall congruence between the tree that we produced after removing lab-associated and extremal variants (except 11083, see above) and the COG-UK tree (Fig 12C). Here, the sample size is much larger, 4172, allowing for a much more quantitative comparison. The correspondence between the two trees is very high with normalized entropy weighted total distance of just 0.12. Because lab-associated and extremal variants were used in the COG-UK tree but not in our tree, this consistency among topologies supports our assertion that the effect of lab-associated and extremal variants will typically not result in large-scale reorganizations of large clades across the phylogeny. Each tree, including our Nextstrain “consensus”, is available for visualization through the UCSC Genome Browser (Fig 8, S6).

### Powerful tools for visualizing and interpreting differences among phylogenies

Different analysis goals require varying levels of phylogenetic resolution and certainty, and it is very likely that hundreds of partially independent phylogenies will be produced studying SARS-CoV-2 evolution. For that reason, we have sought to provide the community with effective methods for tree-based comparisons. In particular, here we provide (1) improved methods for quantitative comparison among trees at the level of whole topologies and at individual nodes; (2) an extremely rapid tanglegram clade rotation method for visualization of differences among tree topologies; and (3) dynamic tree visualization capabilities within the SARS-CoV-2 Genome Browser. Importantly, each method that we present scales well to thousands of samples, and is integrated into the SARS-CoV-2 Genome Browser to facilitate rapid comparison with existing phylogenetic datasets, and to cross-reference sites to molecular information relevant to basic biology, diagnostics, and therapy. Software to run each analysis is available from https://github.com/yatisht/strain_phylogenetics.

## Conclusion and outlook

The SARS-CoV-2 pandemic has driven an impressive global community response providing real-time sequencing data to trace the viral outbreak [2–6]. Because these efforts are both decentralized and urgent, there is potential for systematic differences in data generation and processing to introduce artefactual biases and signal into these data [24]. Similarly, thousands of distinct and differing phylogenies will be made from these data. In this work, we sought to provide tools to detect and interpret sources of conflict and uncertainty in local and global phylogenies. We integrate these into powerful visualization systems to facilitate continued global analysis of viral population dynamics.

## Methods

### Obtaining Nextstrain trees and genotype data

We have downloaded genomic variation data from http://nextstrain.org/ncov, which is ultimately processed and derived from the GISAID database [76], and transformed it into a VCF file with genotypes for all samples as assigned by Nextstrain and a Newick tree file, and associated files for display in the UCSC SARS-CoV-2 Genome Browser. Software to perform this is described here: https://github.com/ucscGenomeBrowser/kent/blob/master/src/hg/utils/otto/nextstrainNcov/nextstrain.py.

### Obtaining and correcting sample metadata

We obtained the GISAID metadata table in bulk from GISAID [77]. Before we were able to search for lab-associated variants, we identified various errors in GISAID metadata files, most of which appear to be due to misspellings and inconsistent naming conventions of “originating” and “submitting” labs across separate sample submissions. We therefore developed a simple approach to detect these errors systematically based on the character content and length of “originating” and “submitting” lab names (https://github.com/lgozasht/COVID-19-Lab-Specific-Bias-Filter). We merge coincident metadata under consistent lab names if “originating” or “submitting” lab names share 70% length similarity and 90% character similarity or 70% length similarity and 80% identical character positions, and output a revised metadata file. We checked all merged names by hand to ensure accuracy, and we maintain a log of each merger event and annotate low confidence mergers. Our updated metadata table is available from https://github.com/lgozasht/COVID-19-Lab-Specific-Bias-Filter.

### Identification of highly recurrent mutations

To detect mutations that reoccur many times through viral evolution, we computed the parsimony score [47,48] for each polymorphic site (our program is available from https://github.com/yatisht/strain_phylogenetics). Briefly, conditional on a tree, we compute the minimum number of branches that have experienced a mutation at a single site to accommodate the phylogenetic distribution of the mutant and reference allele. We note however that recurrent mutations and systematic errors can adversely impact the process of tree building itself, which in turn can impact the inference of parsimony scores: these should therefore be interpreted with caution. This software also computes the sizes of subclades containing the alternative allele after mapping mutations on to the phylogeny using parsimony. This is done by counting the number of descendants following a mutation on a branch that shares the derived allele. These values are reported in *e*.*g*., S1 Table, Fig 1.

### Automated identification of extremal sites

After computing the parsimony score for each polymorphic site, we identified a set of extremal sites that displayed exceptional parsimony scores relative to their allele frequencies as follows. First, we excluded sites with rare alternate alleles, *i*.*e*., sites whose alternate allele frequency was found to be lower than a certain threshold K, where K is the maximum alternate allele frequency at which at least two sites had saturated parsimony scores (*i*.*e*., parsimony score equals alternate allele count). Second, we extracted sites whose parsimony score was found to be the highest among sites with the same or smaller alternate allele frequency. Finally, we also required that extremal sites have an alternate allele frequency that is lowest among all sites with its parsimony score or higher. A program to perform this search is available at https://github.com/yatisht/strain_phylogenetics. This program also optionally allows for extremal sites to be identified without including C>U mutations as these are particularly abundant in SARS-CoV-2 genomes.

### Discovery of lab-associated variants

We systematically flagged possible variants resulting from lab-specific biases based on the proportion of lab-specific alternate allele calls and respective alternate allele frequency (https://github.com/lgozasht/COVID-19-Lab-Specific-Bias-Filter). To do this, we first filtered variants with parsimony score greater than 4 using concurrent Nextstrain tree and VCF files from 4/19/2020. Next, we obtained metadata for all COVID-19 genomes on GISAID (accessed 4/28/2020) and computed the proportion of alternate allele calls contributed by each “originating lab” and “submitting lab” for each filtered variant. We then employed a Fisher’s exact test associating the number of major and alternate alleles attributed to each specific “originating” and “submitting” lab and the respective global major and alternate allele counts. We flagged variants for which one lab accounts for more than 80% of the total alternate allele calls and for which a Fisher’s Exact Test suggests a strong correlation (at the p < 0.01 level) between that lab and samples containing the alternate allele. We note that these cutoffs are somewhat arbitrary, and may require modification in the future, but the subdivision of the data is consistent with our expectations as described in Results. Because samples are not independent and identically distributed, p-values may not reflect error but rather relatedness among samples sequenced at a single facility. For example, if a single lab sampled a particular transmission chain, many chain-specific mutations could be strongly associated with that facility. These should be interpreted cautiously, however, there is no obvious reason why unrelated samples sequenced at the same facility should share an excess of homoplastic mutations. More recent updates to the methods for identifying putative systematic errors can be found at https://virological.org/t/issues-with-sars-cov-2-sequencing-data/473/13, and we also give a short description in the Results/Discussion section.

### Testing for overlap with ARTIC primers

To compare our highly recurrent mutations to the ARTIC primer set, we downloaded the positions of the ARTIC primer binding sites from https://github.com/artic-network/artic-ncov2019/blob/master/primer_schemes/nCoV-2019/V3/nCoV-2019.bed (last accessed 5/6/2020). We computed the number of mutations in each category that overlapped primer binding sites, and we computed the mean distance between each variant and the nearest primer binding site. To test for enrichment for overlap and proximity to primer binding sites, we performed a permutation test where we selected positions at random without replacement across the viral genome to compare to our observed distribution for the real mutations. Each permutation was performed 10,000 times.

### A clade comparison method using branch splits

Comparison of clades is made using a symmetric notion of clades called **splits** as defined in TreeCmp [64]. In a rooted tree, the branches are directed to point away from the root, and a directed branch defining a clade divides all the leaves (lineages) into two categories: those in the clade (reachable by following additional directed edges forward from the branch; we call this being “inside” the branch) and the rest, *i*.*e*., those not in the clade (“outside” the branch; we might say these samples are in the “unclade”). It is the root of the tree that polarizes each split by providing a direction for the branch; *i*.*e*., providing a concept of “inside” versus “outside”, or equivalently “clade” versus “unclade”. For a branch containing a polytomy, the descendant leaves of each of its children form its “clade”. The two sets “clade” and “unclade”, denoted by A and B, define the split. The split is denoted as A|B.

Two phylogenetic trees are similar if their branches produce a similar set of splits. When comparing two phylogenetic trees, we begin by finding the common leaf set: that is, the set of leaves (lineages) that are included in both trees. Then for each tree and each branch in that tree, the **reduced split** is obtained from the split by removing all samples not in the common leaf set for the two trees being compared. To compare two reduced splits, A|B and X|Y, we first compute the size of the set-theoretic symmetric difference between the clades A and X, *i*.*e*., the number of samples that are in A but not in X (denoted by |A\X|), plus the number of samples that are in X but not in A (denoted by |X\A|). This number is denoted by s(A|B,X|Y) and is called the **split distance** between the reduced splits A|B and X|Y. Symbolically,

s(A|B,X|Y) = |A\X| + |X\A|.

The same comparison of B with Y is not necessary as it will yield the same number as obtained by comparing A and X.

Now, if b is a branch in tree T and A|B is its reduced split, the **matching split distance** of the branch b in tree T’ is

c(b,T,T’) = min s(A|B,X|Y) over all reduced splits X|Y in T’.

Given the reduced split A|B for a branch b in a tree T and the set of all reduced splits in a second tree T’, *i*.*e*., {X|Y: X|Y is a reduced split in T’}, the set of **best-matching splits** for A|B is in T’ is defined as

M(b,T’,T’) = {X|Y: X|Y is a reduced split in T’ and s(A|B, X|Y) = c(b,T,T’)}.

That is, every reduced split in M(b,T,T’) has a split distance from A|B equal to the matching split distance of branch b in T’, which is the smallest distance possible. The branches corresponding to the best matching splits are called **best matching branches**.

We can also define the (Shannon) **entropy** of the branch b in the tree T as the entropy in units of bits of its reduced split A|B. Let p = |A|/(|A|+|B|) where |S| denotes the cardinality of a set S. Then the entropy of b is

H(b) = —p log_2_(p)—(1-p) log_2_(1-p).

The proportional entropy weight of the branch b in the tree T is the normalized entropy

w(b) = H(b)/Z, where Z = sum of H(b”) over all branches b” in T.

The **entropy-weighted matching split distance** to tree T’ of branch b in tree T is then

d(b,T,T’) = w(b) c(b,T,T’).

We define a distance measure, called **entropy-weighted total distance**, for two trees T and T’, as the sum of entropy-weighted matching split distance for all branches in T:

D(T,T’) = sum of d(b,T,T’) over every branch b in T.

As this distance measure is not symmetric, we also define a **symmetric** version of it as

S(T,T’) = ½ (D(T,T’) + D(T’,T)).

Since the above metric scales with the size of trees being compared, we also define a **normalized** version using the expected distance [78] (which is computed using trees T_p_ and T_p_’ that randomly permute the leaves of T and T’, respectively, while maintaining the tree structure) as

S_p_(T,T’) = (D(T,T’) + D(T’,T)) / (D(T,T_p_’) + D(T’,T_p_)).

Code for computing these distance measures can be found at https://github.com/yatisht/strain_phylogenetics. This code has additional features, such as the ability to replace the Shannon entropy—p log_2_(p)—(1-p) log_2_(1-p) with related weighting functions such as 2 min{p,1-p}. We find that the method is robust to such replacements.

### Clade orientation for tree comparison

While node rotation algorithms in the context of tanglegram visualization have been implemented in the cophylo and Dendroscope3 tools [70,71], we found these algorithms to be either too slow or inadequate for the large SARS-CoV-2 phylogenies that we compared. We implemented a simple node rotation heuristic, RotTrees, that works well and completes in reasonable time (~1 min) for SARS-CoV-2 trees with ~5K leaves. The algorithm RotTrees accepts two trees, T and T’, each pruned to contain only the shared set of leaves, as input. First, while maintaining the leaf order of T, RotTrees makes a breadth-first traversal in T’, rotating the children of each traversed node based on its average rank (*i*.*e*. child with a lower average rank appears higher), which is the average of the positions of the appearance of that child node's leaves in T. Second, RotTrees repeats the above to rotate the leaves of T while maintaining the leaf order of T’. The previous two steps are repeated until convergence (no new rotations in that iteration) and the final tree rotations for T and T’ are returned. We made this routine available in https://github.com/yatisht/strain_phylogenetics. This may not be optimal for all tree co-visualization purposes, but here we find that this approach is sufficient to produce improved tree visualizations compared to many available packages.

### Phylogenetic trees

We obtained the phylogenetic tree hosted by Nextstrain (accessed 4/19/2020) and used this in our comparisons of clades among trees and as our primary data object for examining apparently recurrent mutation on the tree. We did separately confirm that most apparently recurrent mutations are recovered on the trees produced on different days by Nextstrain.

For comparison of clades among different tree-building approaches, we obtained variant datasets, and phylogenies from Nextstrain (https://nextstrain.org/ncov accessed 4/19/2020-4/26/2020) and from COG-UK (https://cog-uk.s3.climb.ac.uk/20200424/cog_2020-04-24_tree.newick, accessed 4/24/2020).

### Phylogenetic reconstruction

From the 04/19 Nextstrain tree, we created a “reference phylogeny”using IQ-TREE 2 [59,79] to build phylogenies from each of these alignments using the GTR+G nucleotide substitution model. For all other phylogenies, we altered the input by removing or “masking” individual sites, then produced phylogenies from these altered alignments using the same IQ-TREE 2 parameters.

The likelihood of a tree given the alignment from which it was constructed was automatically calculated by the IQ-TREE command used above (*iqtree -s <alignment*.*phy> -m GTR+G*). However, to compute the likelihood of a particular alignment given a different tree, we used the command *iqtree -s <alignment*.*phy> -te <phylogeny*.*nh> -m GTR+G*.

To generate our final tree having masked lab-associated and extremal variants, we used the same command but also included the ultrafast bootstrapping option “-bb 1000” to assist with quantifying uncertainty in our final phylogeny [60]. Finally, we collapsed all branches that were not supported by at least one mutation using parsimony to identify nodes that experienced a mutation.

### Systematic error addition experiments

To investigate the effects of lab-specific alleles on phylogenetic topology, we also introduced artificial errors at control sites. We chose three sites at which to introduce these errors: A11991G, C22214G, and C10029U. To introduce an error, we manually changed a reference allele to an alternate allele for a given sample at a given site. For each of these sites, we chose 10, 25, and 50 samples for which we introduced errors. To mimic the effects of a lab-specific allele, we ensured that each set of samples with artificial errors must come from the same country. We chose Australia due to its high representation in the Nextstrain trees (372 samples in the 04/19 Nextstrain tree). To further mimic lab-specific behavior, we separately introduced errors at the same sites for 10, 25, and 50 randomly selected French samples collected between March 1 and March 17. After introducing these errors, we constructed phylogenies from the modified alignments using IQ-TREE 2 [59,79] as described above. In total, we produced 54 phylogenies in this experiment, introducing errors at three sets of random samples for each of the three sites, at 10, 25, and 50 samples each, for Australian and French samples.

We also repeated this experiment, but introducing errors at pairs of sites simultaneously rather than at individual sites (i.e., A11991G and C22214G, A11991G and C10029U, and C10029U and C22214G). We used the same randomly chosen sets of French samples for this aspect of the experiment, and produced phylogenies by the same methods. In total, we produced 27 phylogenies in this experiment, introducing errors at three sets of randomly chosen samples, at 10, 25, and 50 samples each, for each of the three pairs of sites.

### Comparisons across Nextstrain trees

To understand commonalities in tree structure over time, we used MDS of a distance matrix of normalized entropy-weighted total distances among Nextstrain trees (pruned to 468 shared samples) spanning from March 23 to April 30. To do this, we used the cmdscale() function in base R (https://www.R-project.org/). We computed the correlation between our distance matrix and the proportion of samples shared among topologies produced each day using a Mantel test implemented within the ade4 package in R.

### Producing a Nextstrain consensus tree

To produce a Nextstrain consensus tree, we first pruned all Nextstrain trees to a common set of samples included in each tree. We then used the sumtrees script within the dendropy package [72] to produce a majority rule consensus tree out of each tree requiring at least 50% of trees support a clade for inclusion in the final consensus tree. Specifically, we used the sumtrees function to perform this task. In our cases, that is equivalent to requiring at least 16 of 31 trees contain a given clade to include it.

## Supporting information

S1 Text High Allele Frequency Variants Could Reveal Cross-Contamination. (DOCX)

S2 Text Potential for Correlated Error in Our Dataset. (DOCX)

S3 Text Entropy Weighted Distance is a Robust Tree-Distance Measure. (DOCX)

S4 Text Step-by-step instructions for setting up a genome browser session with a custom tree and VCF. (DOCX)

S1 Fig Alternate allele count versus parsimony score for the Nextstrain 4/20/2020 dataset and tree. Each point is labeled as in Fig 2A with additional extremal points annotated. The dashed line is fit to the extremal points and has log2-base slope 3.518.
(PNG)

S2 Fig Lab-associated variants influence tree topology. Phylogenies created using the variants from 04/19 Nextstrain tree without modification (left) and with lab-associated variants completely masked (right) demonstrate movement of multiple samples between sub-clades. Those samples with the greatest changes in placement between the phylogenies are bolded. This includes many samples containing lab-associated variants that we masked, which are colored in red.
(PNG)

S3 Fig Comparisons between the reference phylogeny, built from the 04/19/2020 release of Nextstrain, to phylogenies built by entirely masking lab-associated variants (blue), control sites (grey), and extremal sites (brown) are shown for Robinson-Foulds **(A)**, Quartet **(B)**, Path Difference **(C)**, and Triples **(D)** scores as calculated by TreeCmp [64]. Horizontal lines indicate scores for phylogenies constructed after masking all lab-associated sites (blue), all control sites (grey), all extremal sites (brown), or using an unaltered Nextstrain 04/19/2020 dataset (black).
(PNG)

S4 Fig The entropy-weighted total distance values between the reference phylogeny and phylogenies constructed after entirely masking all samples with an alternate allele at a given site are shown. The sites used here are the same sites corresponding to lab-specific shown in Fig 5.
(PNG)

S5 Fig Tanglegrams for the two Nextstrain trees released on 04/19/2020 (left) and 04/20/2020 (right). (**A**) Without tree rotation, the tanglegram has a large mesh of connecting lines, making it hard to see the tree correspondence. (**B**) With trees rotated using RotTrees, the tanglegram is more visually appealing and the tree correspondence is a lot clearer.
(PNG)

S6 Fig UCSC Genome Browser display of the trees from Fig 11C (COG-UK tree from 4/24, restricted to 422 samples in common with consensus tree of Nextstrain trees 3/23-4/30, and Nextstrain consensus tree), colored by Nextstrain clade assigned to sample. Interactive view: http://genome.ucsc.edu/s/SARS_CoV2/cogVsNsCladeColors
(PNG)

S1 Table Lab-associated variants discovered in our dataset. (XLSX)

S2 Table Highly recurrent, low alternate allele frequency variants. (XLSX)

S3 Table High alternate allele frequency, highly recurrent sites. (XLSX)

S4 Table Lab-associated, low parsimony score sites. (XLSX)

S5 Table Log-likelihood of outlier alignments based on entropy-weighted total distance. (XLSX)

S6 Table GISAID IDs whose clade annotation changed in the Nextstrain tree from 4/19/2020 to 3/30/2020, and from 4/19/2020 to 4/28/2020. (XLSX)

S7 Table Acknowledgements GISAID Sample ID’s From March 19-March31. (PDF)

S8 Table Acknowledgements GISAID Sample ID’s From April 1-April 15. (PDF)

S9 Table Acknowledgements GISAID Sample ID’s From April 16-April3. (PDF)
