# Supplementary material for: Stability of SARS-CoV-2 phylogenies
Source: PLoS Genet. 2020 Nov 18;16(11):e1009175. doi: 10.1371/journal.pgen.1009175 (PMC7721162; doi:10.1371/journal.pgen.1009175)
Supplement: S1 Text — (DOCX) [file pgen.1009175.s001.docx]

**Text S1. High Allele Frequency Variants Could Reveal Cross-Contamination**

Although much of this work is focused on detecting and characterizing the impacts of low-frequency highly recurrent and lab-associated alleles, cross-contamination among samples is also a potential source of widespread phylogenetically discordant sites that warrants mentioning. The majority of labs performing viral genome sequencing are processing multiple samples. There is therefore a significant possibility for contamination to drive the apparent recurrence of high-frequency mutations.

Unfortunately, contamination, short recombination tracts, and high-frequency recurrent mutations create largely similar predictions about the distributions of recurrent alleles (Table 1). Yet there are some distinctions one might expect. Both recombination and contamination require a sample or lineage to encounter another of a different allele to be observable. Therefore, high-frequency alleles should typically be involved in both recombination and contamination events. Additionally, all else being equal, we expect that we would observe equal numbers of forward and backward mutations across the tree for recombination and contamination, but not for recurrent mutation, which can be quite biased (see above). Consistent with this idea, six out of eleven sites with alternate allele frequency above 10% show evidence of additional forward and backward mutation even after removing lab-associated variants (Figure 2, Table S3), consistent with the presence of contamination or recombination. Hence, even if we could remove all systematic errors, contamination should be considered as a possible source of homoplastic mutation before confident conclusions are drawn about natural selection or the presence of viral recombination.
