# Supplementary material for: Stability of SARS-CoV-2 phylogenies
Source: PLoS Genet. 2020 Nov 18;16(11):e1009175. doi: 10.1371/journal.pgen.1009175 (PMC7721162; doi:10.1371/journal.pgen.1009175)
Supplement: S2 Text — (DOCX) [file pgen.1009175.s002.docx]

**Text S2. Potential for Correlated Error in Our Dataset**

To investigate the potential for highly correlated lab-associated variants in the real data, we extracted the set of mutations with alternate allele counts of 10 or more and where at least 80% of samples containing alternate alleles were derived from a single lab (Table S4). This set of alleles shares many features with sites that we believe to be sites of real variation, suggesting that many are indeed true variants. In aggregate, these mutations are not enriched for proximity to ARTIC primer binding sites (P = 0.9502, permutation test), the C>U mutation fraction is similar to that observed in high-frequency sites (P = 0.5307, Fisher’s exact test), they affect amino acids at similar rates to those of high-frequency alleles (P = 0.7643), and they have low parsimony scores even relative to our “two-error” experiments (1-2, Table S4). Our results therefore suggest that highly correlated lab-associated variants are relatively rare.

It is noteworthy that there is overlap with some of the lab groups who contributed high parsimony score lab-associated alleles (nine out of 24, Table S1). However, these are also groups who contributed the most genome sequences in our dataset and they are therefore the most likely to be associated with low-frequency variation or lab-associated variants for that matter. Most such variants do not overlap in samples with other lab-associated variant sites indicating that if they were independent mutations we would likely see their placement vary across the tree. Nonetheless, in one extreme case, G11417U, U14073C, and A23947G co-occur in a single clade across many samples suggesting that these sites could impact tree-building algorithms even more substantially than those in our two-error simulations. However, samples containing these variants are not unusually divergent relative to the clade size (the average pairwise nucleotide diversity is 1.18 sites/genome) as would be expected if they were incorrectly grouped. Moreover, we emphasize that real variants should be correlated on the viral phylogeny and that these do not constitute sequencing errors. We believe that our approach has likely identified the majority of lab-associated recurrent variants in this dataset that occur in more than a handful of samples.
