# Supplementary material for: Stability of SARS-CoV-2 phylogenies
Source: PLoS Genet. 2020 Nov 18;16(11):e1009175. doi: 10.1371/journal.pgen.1009175 (PMC7721162; doi:10.1371/journal.pgen.1009175)
Supplement: S3 Text — (DOCX) [file pgen.1009175.s003.docx]

**Text S3. Entropy Weighted Distance is a Robust Tree-Distance Measure**

To confirm that our distance measure will be robust and consistent with expectations, we compared the set of all pairwise distances between trees produced by Nextstrain from March 23 to April 30 across a range of tree distance statistics. In particular, we find that entropy-weighted total distance is strongly correlated with quartet, Robinson-Foulds and matching-split tree distance measures (P < 1e-5, in all cases, Mantel test). This strongly suggests that our approach yields robust and interpretable tree distances.
