# Supplementary material for: Stability of SARS-CoV-2 phylogenies
Source: PLoS Genet. 2020 Nov 18;16(11):e1009175. doi: 10.1371/journal.pgen.1009175 (PMC7721162; doi:10.1371/journal.pgen.1009175)
Supplement: S4 Text — (DOCX) [file pgen.1009175.s004.docx]

**Text S4. Step-by-step instructions for setting up a genome browser session with a custom tree and VCF**

We provide researchers the ability to upload their own set of aligned SARS-CoV-2 genomes and an accompanying phylogenetic tree. This allows the researcher to compare their tree to trees from Nextstrain and COG-UK, and to map alleles characteristic of particular clades to sites in the virus genome of functional, diagnostic, or therapeutic significance. Alignment files built by most tools are too large to transfer when the number of viral samples gets large, so we require they be converted to the more compact VCF format with sample genotypes before upload (the [Msa2Vcf](https://lindenb.github.io/jvarkit/MsaToVcf.html) tool produces VCF with sample genotypes). Once the VCF file for the alignment is created, one does the following:

1. Compress the VCF file with bgzip and index it with tabix following the instructions here: <https://genome.ucsc.edu/goldenPath/help/vcf.html>

2. Place the .vcf.gz, .vcf.gz.tbi and a newick format file for the phylogenetic tree (or trees) on a web or ftp server accessible to [genome.ucsc.edu](http://genome.ucsc.edu/). As a hypothetical example, all relevant files could be available from the same server as shown:

https://my.lab.org/my.vcf.gz

https://my.lab.org/my.vcf.gz.tbi

https://my.lab.org/my.newick

3. Replace the example URLs with actual URLs in the following custom track specification line (all one line, no line breaks), copy and paste into the input in <https://genome.ucsc.edu/cgi-bin/hgCustom> (making sure the SARS-CoV-2 genome is selected):

track name=myTreeAndVcf type=vcfTabix visibility=pack hapClusterEnabled=on hapClusterHeight=500 bigDataUrl=https://my.lab.org/my.vcf.gz hapClusterMethod="treeFile https://my.lab.org/my.newick"
