# Supplementary figures and images for: Stability of SARS-CoV-2 phylogenies
Source: PLoS Genet. 2020 Nov 18;16(11):e1009175. doi: 10.1371/journal.pgen.1009175 (PMC7721162; doi:10.1371/journal.pgen.1009175)

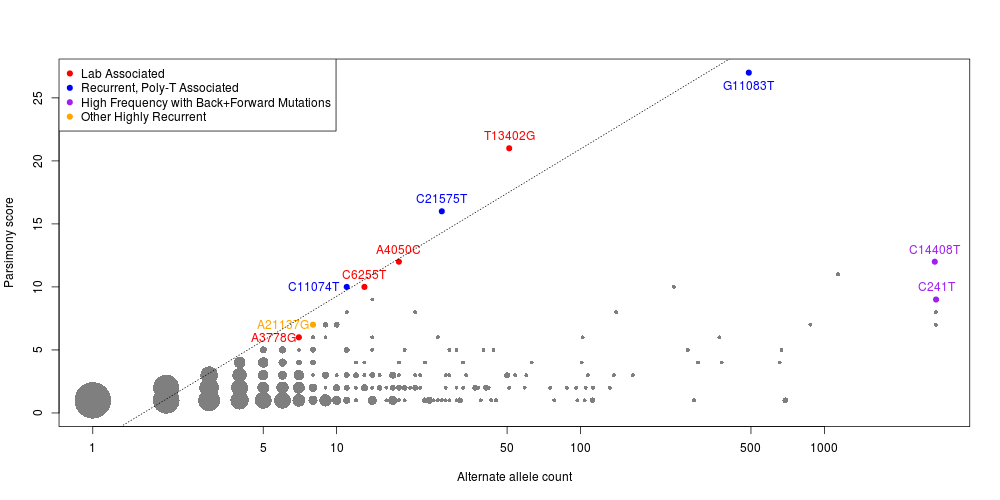

Supplement: S1 Fig — Each point is labeled as in Fig 2A with additional extremal points annotated. The dashed line is fit to the extremal points and has log2-base slope 3.518. (PNG) [file pgen.1009175.s005.png]

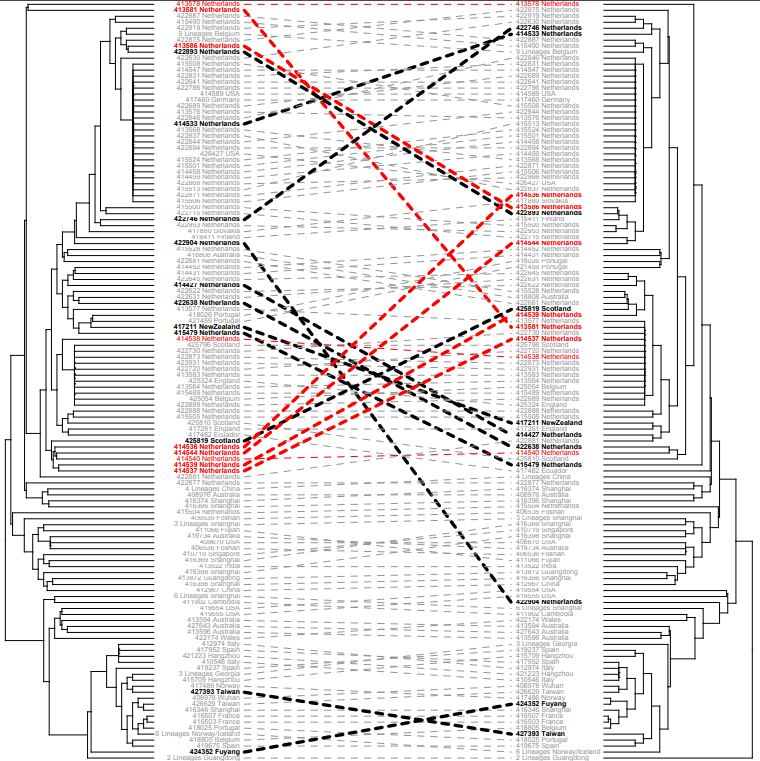

Supplement: S2 Fig — Phylogenies created using the variants from 04/19 Nextstrain tree without modification (left) and with lab-associated variants completely masked (right) demonstrate movement of multiple samples between sub-clades. Those samples with the greatest changes in placement between the phylogenies are bolded. This includes many samples containing lab-associated variants that we masked, which are colored in red. (PNG) [file pgen.1009175.s006.png]

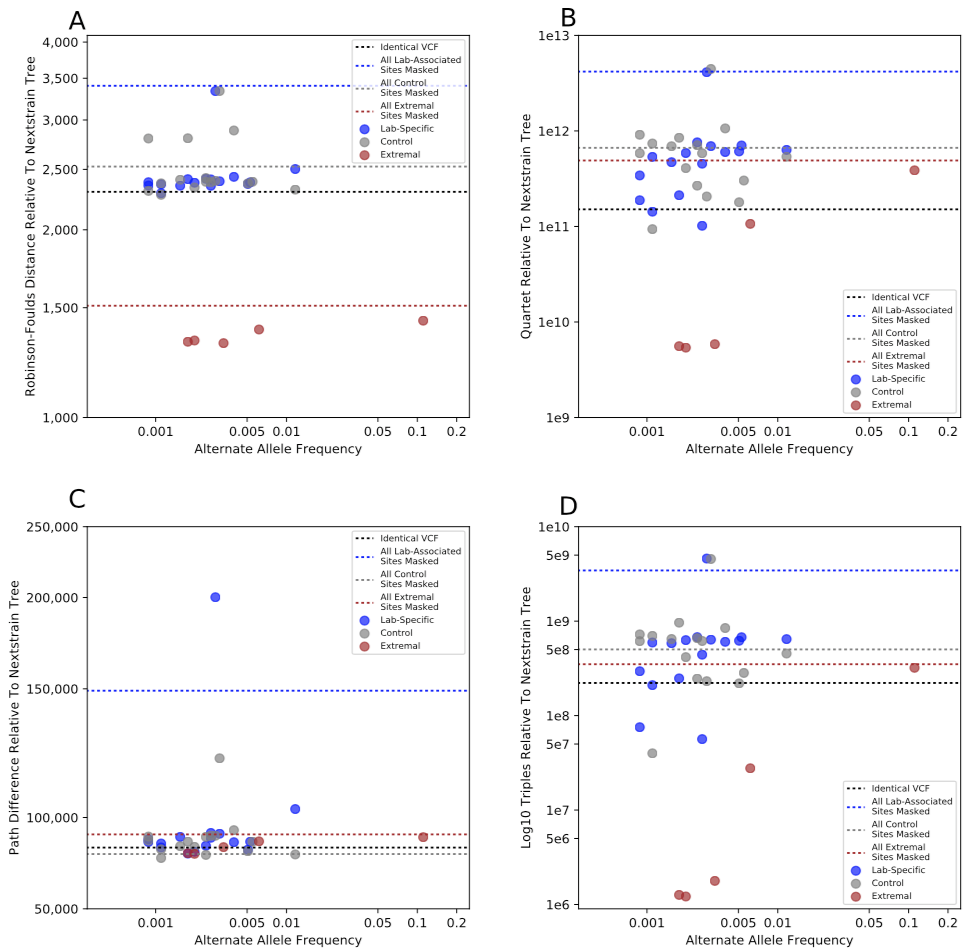

Supplement: S3 Fig — Comparisons between the reference phylogeny, built from the 04/19/2020 release of Nextstrain, to phylogenies built by entirely masking lab-associated variants (blue), control sites (grey), and extremal sites (brown) are shown for Robinson-Foulds (A), Quartet (B), Path Difference (C), and Triples (D) scores as calculated by TreeCmp [64]. Horizontal lines indicate scores for phylogenies constructed after masking all lab-associated sites (blue), all control sites (grey), all extremal sites (brown), or using an unaltered Nextstrain 04/19/2020 dataset (black). (PNG) [file pgen.1009175.s007.png]

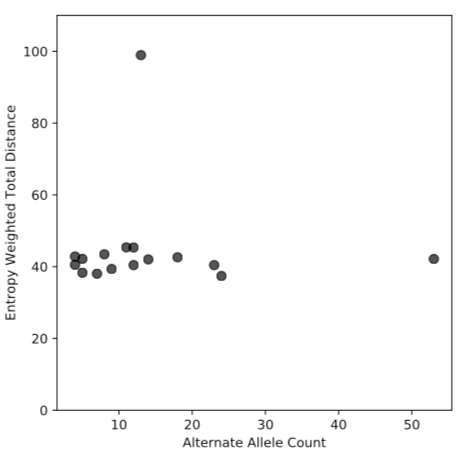

Supplement: S4 Fig — The sites used here are the same sites corresponding to lab-specific shown in Fig 5. (PNG) [file pgen.1009175.s008.png]

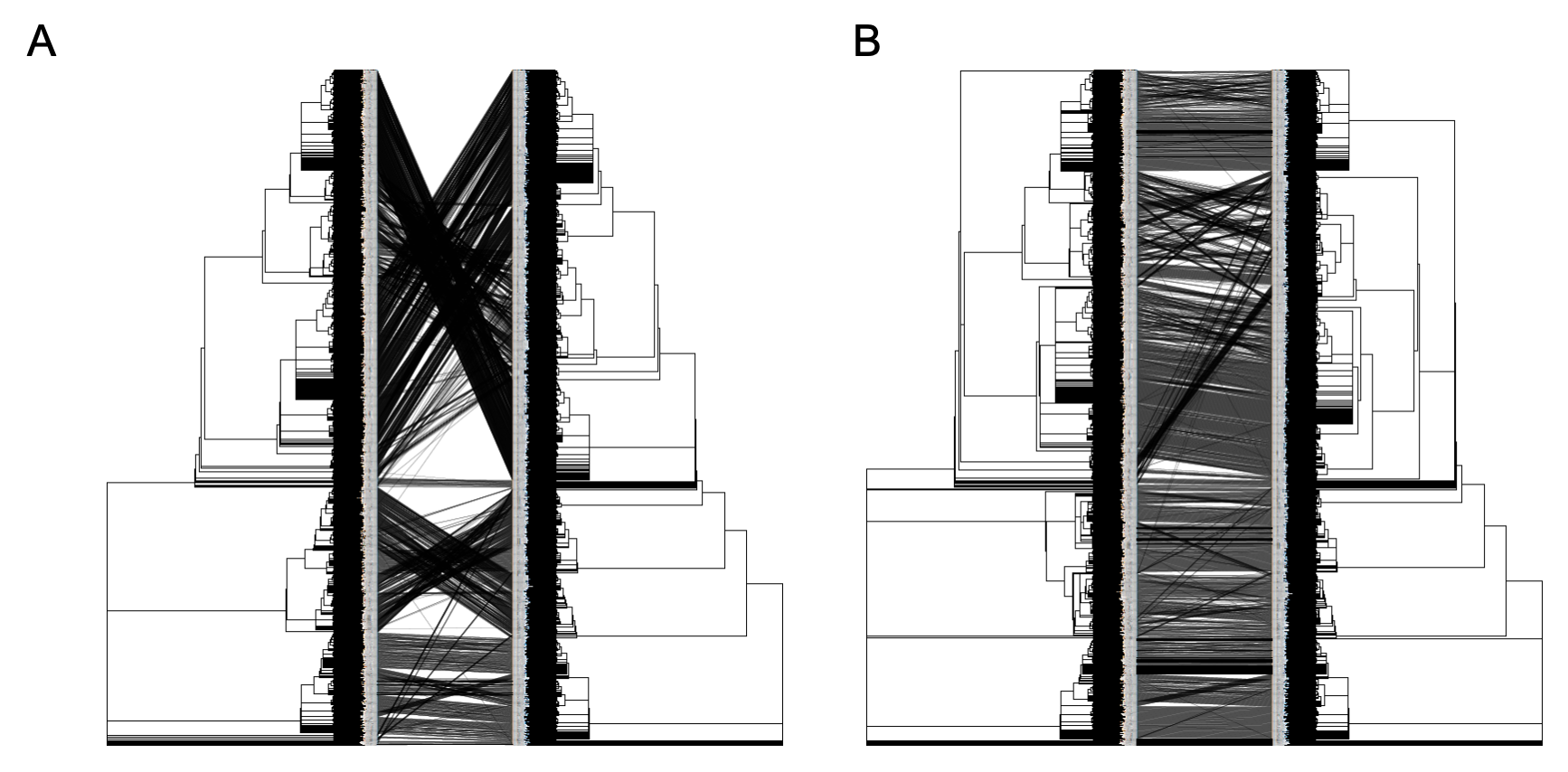

Supplement: S5 Fig — Tanglegrams for the two Nextstrain trees released on 04/19/2020 (left) and 04/20/2020 (right). (A) Without tree rotation, the tanglegram has a large mesh of connecting lines, making it hard to see the tree correspondence. (B) With trees rotated using RotTrees, the tanglegram is more visually appealing and the tree correspondence is a lot clearer. (PNG) [file pgen.1009175.s009.png]

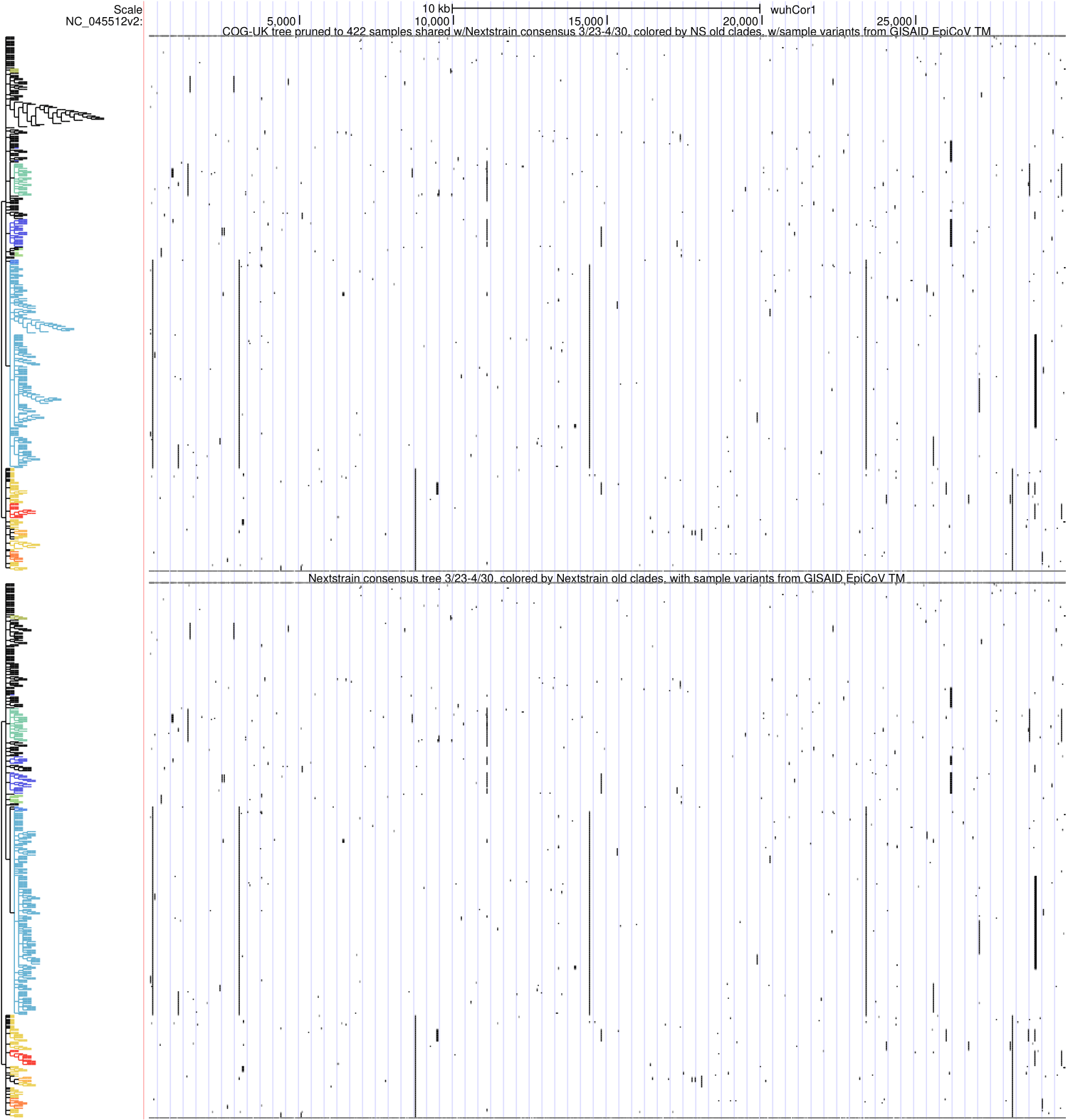

Supplement: S6 Fig — Interactive view: http://genome.ucsc.edu/s/SARS_CoV2/cogVsNsCladeColors (PNG) [file pgen.1009175.s010.png]
